# Skeletal Maturity and Age‐Related Changes in Immune Cells and Circulatory Factors Impair Large‐Scale Bone Regeneration

**DOI:** 10.1111/acel.70177

**Published:** 2025-07-21

**Authors:** Luciana Yamamoto de Almeida, Catharine Dietrich, Ashleigh S. Hanner, Katelyn M. McTighe, Daniel Martin, NIDCD/NIDCR Genomics and Computational Biology Core, Todd Fairbanks, Thomas M. Link, John M. Le, Natasha Curry, Priyam Jani, Xin Gao, Wenli Yu, Francesca V. Mariani, Olivier Duverger, Janice S. Lee

**Affiliations:** ^1^ Craniofacial Anomalies and Regeneration Section National Institute of Dental and Craniofacial Research (NIDCR), National Institutes of Health Bethesda Maryland USA; ^2^ Genomics and Computational Biology Core National Institute on Deafness and Other Communication Disorders (NIDCD)/NIDCR Bethesda Maryland USA; ^3^ Department of Oral & Maxillofacial Surgery University of California San Francisco San Francisco California USA; ^4^ Department of Stem Cell Biology and Regenerative Medicine, Keck School of Medicine University of Southern California Los Angeles California USA

## Abstract

Large‐scale bone defects require complex surgical procedures to repair, but full restoration of the bone is not guaranteed due to the significant tissue loss involved. In contrast, fractures can frequently be treated with conservative techniques. Particularly, ribs have a remarkable ability to spontaneously regenerate large‐scale bone defects. However, we show here that skeletal maturity and age are associated with a decrease in the regenerative potential of human ribs. To investigate skeletal maturity and age‐related cellular and transcriptional changes during large‐scale bone regeneration, we used a mouse model that mimics the regenerative clinical features of human ribs. Unlike immature mice, mature mice lose the ability to regenerate after rib resection, and instead of bone, the resected rib space is repaired with abundant fibronectin cells. In addition, bone repair in mature mice presents reduced immune cell infiltration, which coincides with decreased levels of circulatory pro‐inflammatory factors. To address the role of cell–cell communication and test whether skeletal maturity and age‐related changes in immune cells and circulatory factors influence bone regeneration, we used immunodeficient mouse strains and performed heterochronic parabiosis. In immature mice, defective immune cell function altered callus composition rather than inhibiting bone regeneration. Remarkably, under parabiosis, a systemic pro‐regenerative response is triggered exclusively in resected immature mice and is capable of partially rescuing bone regeneration in mature mice otherwise unable to regenerate spontaneously. Collectively, these findings suggest that therapeutic strategies focused on identifying pro‐regenerative immune factors are promising for supporting the regeneration of large bone defects.

## Graphical abstract

Although the ribs are the only human bones capable of regenerating large‐scale defects, this remarkable ability declines with skeletal maturity and age. Here, we reproduce skeletal maturity and age‐related phenotypic changes in a mouse model of rib resection. Of note, defective immune cell function in immature mice alters the development of bone calluses but does not stop bone regeneration. Conversely, heterochronic parabiosis experiments demonstrated that systemic factors released exclusively after rib resection in immature mice rescue bone regeneration in mature mice that would otherwise be unable to heal large‐scale bone defects on their own. Taken together, these findings suggest that appropriate regenerative local and systemic immune responses must be identified for adequate bone regeneration and treatment of challenging large‐scale bone defects. dpr, days post‐resection.

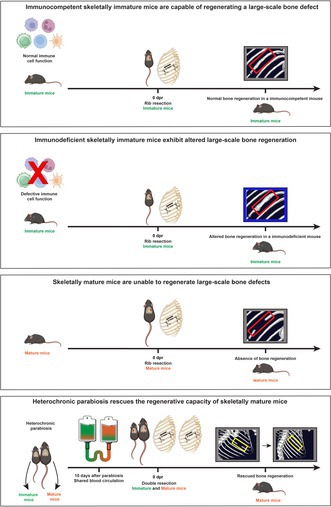

**Abbreviations**
BAFF: B‐cell activating factor
BMD: bone mineral density
BMP: bone morphogenetic protein
CT: computed tomography
DEGs: differentially expressed genes
dpr: days post‐resection
G‐CSF: granulocyte colony stimulating factor
GO: gene ontology
Hh: hedgehog
HU: hounsfield units
IHC: immunohistochemistry
IL: interleukin
IPA: ingenuity pathway analysis
KO: knockout
M‐CSF: macrophage colony‐stimulating factor
NK: natural killer
RNAseq: RNA sequencing
RUNX2: runt related transcription factor 2
scRNAseq: single‐cell RNA sequencing
*Smad4* or SMAD4: SMAD family member 4
SSPC: skeletal stem and progenitor cells
TGF‐β: transforming growth factor‐β
WT: wild‐type
μCT: micro computed tomography

## 1 Introduction

The age‐related decrease in bone healing capacity has been extensively studied in fracture models, pointing to local and systemic changes impacting the microenvironment and function of skeletal stem and progenitor cells (SSPC) (Ambrosi et al. 2021; Baht et al. 2015; Clark et al. 2020; El Khassawna et al. 2017; Gao et al. 2019; Hebb et al. 2018; Josephson et al. 2019; Loi et al. 2016; Mancinelli and Intini 2023; Vi et al. 2018; Wagner et al. 2019; Zhang et al. 2022). In most cases, the human ability to spontaneously repair bones that restore anatomical alignment and functional rehabilitation is confined to minor injuries. Conversely, large bone defects or deformities (> 1 cm^3^) caused by congenital or acquired etiology cannot heal spontaneously, and bone graft surgery remains the leading treatment for reconstruction (Schemitsch 2017). Ribs are frequently used as grafts for autologous transplantation and represent a particular exception in terms of their intrinsic ability to self‐heal large skeletal segments in donor areas (Munro and Guyuron 1981; Tripuraneni et al. 2015). Thus, this unique regenerative capacity of the rib may provide an excellent model to investigate the effects of skeletal maturity and aging on postnatal regeneration of large‐scale bone defects.

Large‐scale rib defects cannot undergo self‐repair unless the periosteum is present (Tripuraneni et al. 2015). Given that it offers a supportive environment with cellular and biological components, the periosteum is essential for skeletal development and repair (Matthews et al. 2021; Roberts et al. 2015). Importantly, recent studies indicate that the periosteum contains a variety of SSPCs that contribute to cartilage and bone formation (Colnot 2009; Duchamp de Lageneste et al. 2018). Among these, *Sox9* ^+^ SSPCs have been identified with hybrid osteochondral properties that orchestrate large‐scale rib regeneration in mice by recruiting other cell types to the resected area to support bone repair (Kuwahara et al. 2019; Serowoky et al. 2022). Furthermore, when the bones are injured, the inflammatory response initiates, which helps with SSPC differentiation, neovascularization, and subsequent repair (Loi et al. 2016). It has been demonstrated that the involvement of immune cells is essential in the early inflammatory stage for an effective bone healing process (El Khassawna et al. 2017; Mancinelli and Intini 2023; Rapp et al. 2016; Schlundt et al. 2018; Zhang et al. 2021, 2022). However, age‐related factors at the cellular and molecular level in the callus environment may interfere with the well‐orchestrated process of bone repair and cause delayed union or non‐union of fractures, which can impact the clinical outcomes of elderly patients (Foulke et al. 2016; Mancinelli and Intini 2023). Age‐related chronic inflammation has been implicated as a key factor in SSPC dysfunction (Ambrosi et al. 2021; Josephson et al. 2019; Loi et al. 2016; Mancinelli and Intini 2023). Accordingly, in vivo experimental studies have previously demonstrated that the macrophage profile changes with age, which has an impact on their ability to heal fractures (Clark et al. 2020; Löffler et al. 2019). Furthermore, although lymphocytes regulate the function of osteoblasts and osteoclasts, especially during the remodeling phase of fracture healing (Zhang et al. 2021), increasing age is associated with changes in their production and maturation that can negatively affect bone repair in elderly patients (Clark et al. 2017). On the other hand, heterochronic parabiosis and bone marrow transplantation experiments have shown that circulatory factors in the bloodstream of young mice can improve fracture healing in old mice (Baht et al. 2015; Vi et al. 2018; Zhang et al. 2022).

Here we demonstrate that although the rib has a remarkable regenerative capacity even when subjected to large surgical resection, this potential decreases with age in humans. Importantly, we also used an animal model with mice whose age range reflects their skeletal maturation states and plays a significant impact on their regenerative capabilities. This mouse model recapitulates human clinical features and reveals new insights into the skeletal maturity and age‐related changes in SSPCs, immune cells, and circulatory factors that are associated with altered callus formation. Of note, we demonstrate that circulatory factors with regenerative properties are exclusively released into the blood of immature mice in response to rib resection and can reverse impaired regeneration in mature mice under heterochronic parabiosis. Elucidating the fundamental molecular and cellular differences that lead to skeletal maturity and age‐related changes in the ability to repair large‐scale rib defects will result in better strategies for treating challenging bone defects.

## 2 Results

### 2.1 Large‐Scale Rib Regeneration Declines With Age in Humans

**FIGURE 1:**
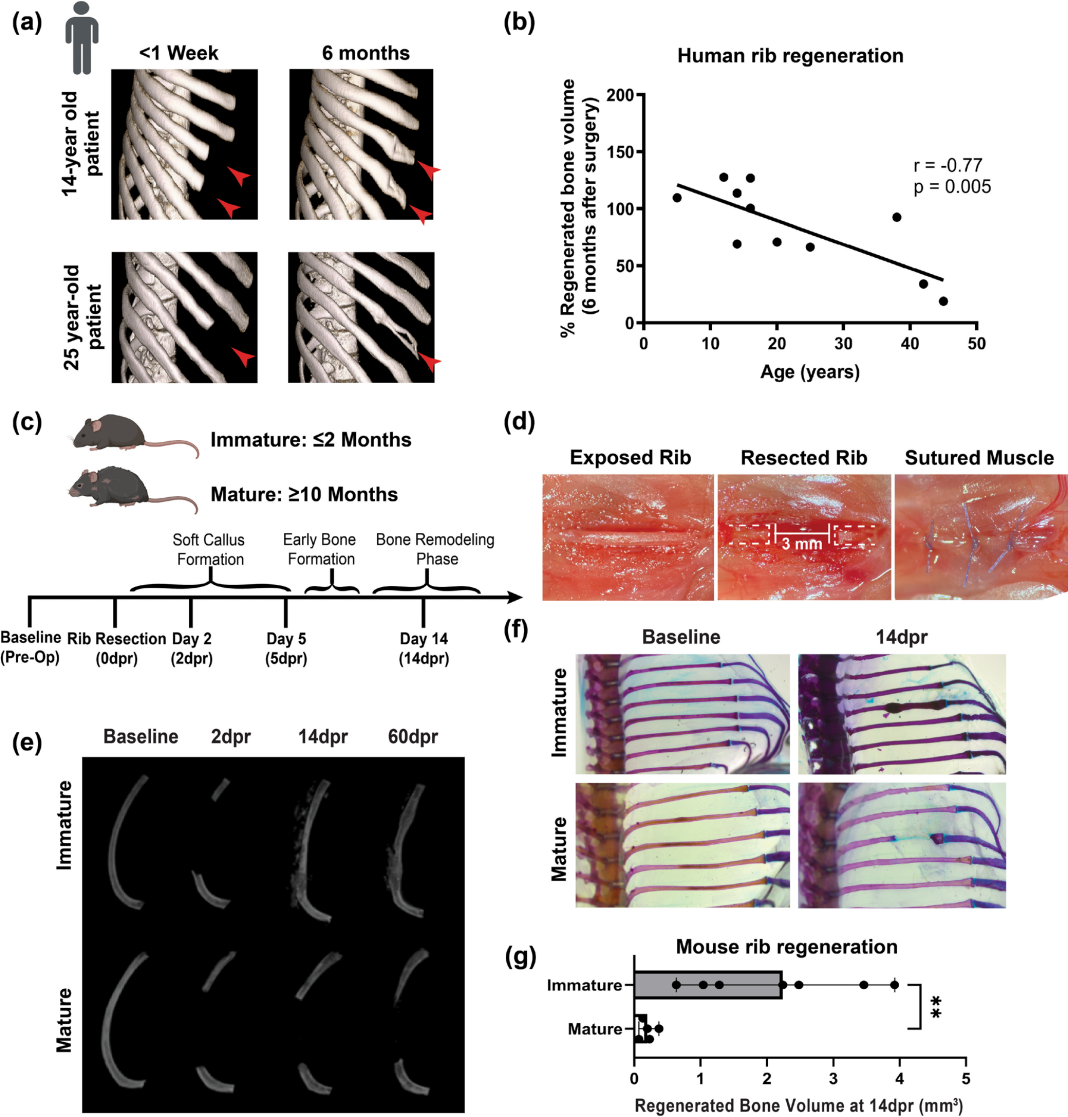
Age decreases the regenerative capacity of large‐scale rib defects in humans, which is recapitulated in a mouse model. (a) Representative CT images of the chest of patients aged 14‐ and 25‐years undergoing rib resection surgeries for autologous transplantation and longitudinally monitored to assess changes in rib regeneration (red arrowheads) between < 1 week and 6‐month post‐resection. (b) Assessment of individual variation in the percentage of bone regeneration at 6 months revealed a negative correlation with age. The association between age and bone regeneration outcomes was analyzed by the Pearson correlation coefficient (*r* = −0.77 and *p* = 0.005, *n* = 11). (c) The timeline represents the sequence of events following rib resection. (d) Surgical sequence for a typical large‐scale rib resection technique. The dashed white lines represent the ends of the after the resection surgery generating a 3 mm rib bone defect. (e) Representative image of pre‐ (baseline) and postoperative μCT reconstructions showing bone regeneration in an immature mouse (≤ 2‐months) from 14 to 60dpr, while the mature (≥ 10‐months) mouse never fully regenerated the rib. (f) Whole‐mount skeletal staining using Alcian Blue and Alizarin Red to identify chondroid and mineralized tissue, respectively, before and 14dpr. Note that Alizarin red is absent in the rib‐resected site of the mature mouse. (g) Quantification of the regenerated bone volume (mm^3^—**p < 0.01 at 14dpr; *n* = 5–7 mice/group); all data represent the median of individual values and median with 95% confidence interval (CI). Statistical comparison was made via the unpaired *t* test.

To determine whether the human potential for regeneration of large‐scale rib defects correlates with age, we first examined 12 patients with a craniofacial bone defect or congenital deficiency who underwent rib resection surgeries for autologous transplantation and consented to participate in a University of California, San Francisco (UCSF) Institutional Review Board approved study. After rib resection, 11 patients (16 ± 13.35 years, range 5–45, 4 females and 7 males—Table S1) were monitored longitudinally using computed tomography (CT) imaging. The CT images obtained immediately postoperatively (1 week following surgery) were compared to those taken at 3‐ and 6‐month postoperative times. Using linear regression analysis, we found that age correlates with bone regenerative capacity (Figure 1a,b). The older the patient, the lower their regenerative capacity for spontaneous bone formation. These findings indicate that the large‐scale regenerative capacity of ribs declines with age in humans.

### 2.2 A Large‐Scale Rib Resection Mouse Model Recapitulates the Age‐Related Decline in Human Regenerative Capacity

To identify potential cellular and molecular processes associated with the decline in bone regeneration, we first tested whether the ability of ribs to self‐heal decreases with skeletal maturity and age in a previously developed mouse model that mimics large‐scale rib regeneration in humans (Kuwahara et al. 2019; Tripuraneni et al. 2015; Yamamoto de Almeida et al. 2024). A detailed overview of the experimental timeline is shown in Figure 1c. The details of the surgical steps of rib resection in mice are shown in Figure 1d.

Briefly, resection was performed, generating 3 mm rib bone defects, and micro computed tomography (μCT) imaging was acquired at baseline (before rib resection), 2, 14, and 60 days post‐resection (dpr), allowing us to capture the pre‐ and postoperative state, bone regeneration, and complete bone formation. Young skeletally immature mice (≤ 2‐months, hereafter referred to as immature) show bone regeneration as early as 14 days after surgery, whereas middle‐aged skeletally mature mice (≥ 10‐months, hereafter referred to as mature) never fully regenerated the rib even after 60dpr (Figure 1e). At 14dpr, the resected rib spaces of immature mice were repaired with newly mineralized tissue, as evidenced by Alizarin Red staining, whereas the repaired area of mature mice shows a light Alcian blue staining, which is suggestive of chondroid tissue (Figure 1f). Although there is variability in bone regenerative capacity between individual immature mice, measurement of bone volume at the resection site confirmed that it was significantly suppressed in mature mice (Figure 1g). Thus, these results suggest that the bone regenerative capacity of mice declines with skeletal maturity and age, recapitulating the repair of large rib defects in humans.

### 2.3 Reduced Expression of Osteochondrogenic Genes and the Disorganized Spatial Distribution of SOX9 and SP7 Are Associated With Impaired Rib Regeneration

**FIGURE 2:**
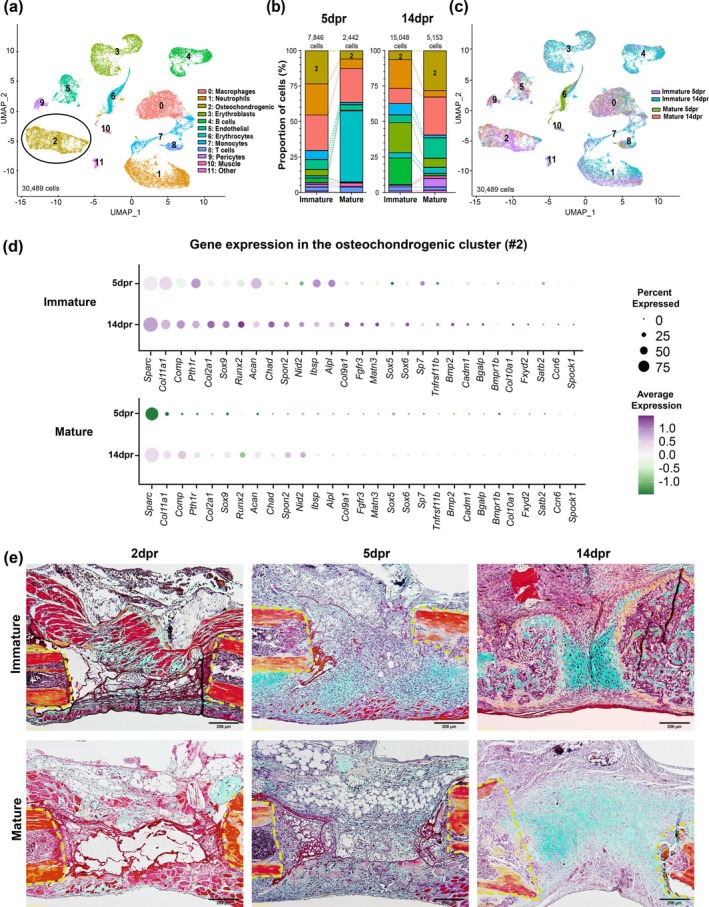
Single‐cell RNA sequencing and histological analysis reveal skeletal maturity and age‐related changes in the repair callus niche of large‐scale rib defects. (a) Uniform Manifold Approximation and Projection (UMAP) visualization of 12 (#0–11) major cell clusters identified by single‐cell RNA sequencing (scRNAseq) data in the large‐scale rib defect repair callus niche of immature and mature mice at 5 and 14dpr. The osteochondrogenic cluster (#2) is delimited by a circle. (b) Bar graphs showing comparison of relative proportions of cells in repair calluses from immature and mature mice at 5 dpr (*n* = 2 mice/group) and 14dpr (*n* = 2 mice/group). (c) UMAP visualization of cell clusters identified by scRNAseq data in the callus repair niche of large‐scale rib defects of immature and mature mice at 5 and 14dpr showing both ages and time points separately. Clusters were generated using a resolution of 0.1. Osteochondrogenic and immune cells were annotated using a combination of reference‐based (Mouse Cell Atlas http://bis.zju.edu.cn/MCA/) and manual annotation as described in Table S2. (d) Dot plot from scRNAseq of repair calluses showing age‐related changes in the expression of genes relevant for cartilage and bone formation in the osteochondrogenic cluster (#2). Percentage of gene expression is represented by the area of a circle, and gene expression levels are color‐coded, ranging from purple (high expression levels) to green (low expression levels). Expression values are normalized and scaled averages. (e) Movat‐Pentachrome staining at the rib resection site at 2, 5 and 14dpr. Mineralized tissue is visualized in yellow/orange and chondroid tissue in light blue to dark green. The dashed yellow lines represent the ends of the ribs after resection surgery. Scale bars: 200 μm.

As periosteum‐based bone regeneration only occurs under specific age‐related biological conditions that favor the stimulation of reparative responses, we sought to investigate the cellular and molecular factors that coordinate and lead to bone regeneration in immature mice but not in mature ones. Gene Ontology (GO) enrichment analysis from bulk RNA sequencing (RNAseq) data collected from callus tissues at 0 and 2dpr identified extracellular matrix reorganization, bone development, and cellular differentiation as significantly different biological functions involved in the early stages of bone repair in immature versus mature mice (Figure S1a,b). Indeed, at 2dpr, genes encoding the transcription factors SP7, also known as Osterix, and runt‐related transcription factor 2 (RUNX2), which are crucial players for the early stages of osteoblast differentiation, were identified as being significantly changed in expression in immature versus mature mice (Figure S1c). We further analyzed transcriptional expression at the single‐cell level (single‐cell RNA sequencing—scRNAseq), which revealed a lower overall degree of cellular heterogeneity and proportion of osteochondrogenic cells (#2) with a decreased relative expression of osteogenic and chondrogenic genes in the callus from mature compared to immature mice at 5dpr (Figure 2a–d; Table S2). This is consistent with the histological findings at 5dpr, in which mesenchymal condensation of soft callus followed by chondrogenic differentiation and maturation was observed at the injury site of immature mice, as evidenced by the areas containing hypertrophic chondrocytes stained with Alcian blue, whereas mature mice exhibited fibrin‐rich granulation tissue and abundant adipocytes (as shown in Figure 2e). We were unable to identify adipocytes using scRNAseq, likely due to the combined limitations of the sample preparation steps and technical resolution.

**FIGURE 3:**
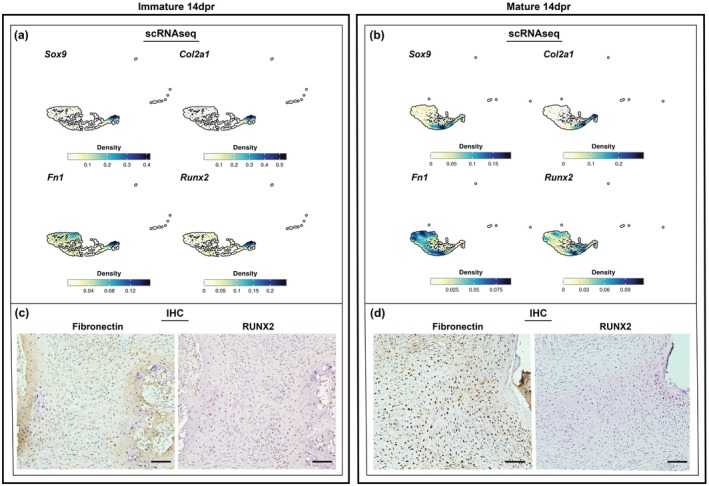
The repair region of large‐scale rib defects in mature mice is mainly occupied by fibronectin‐expressing cells. Nebulosa density plots visualization of the relative imputed expression of indicated transcript based on similarity in the osteochondrogenic cluster (#2) of immature (a) and mature mice (b). Color intensity represents the expression level of each gene. Each Nebulosa plot shows data with an independent scale (lower to maximum), reflecting different measurement ranges that were adjusted to maximize the visibility of these distinct populations. Immunohistochemical representation of fibronectin‐positive and RUNX2‐positive cells in the repair calluses of immature (c) and mature mice (d) at 14dpr. Scale bars: 50 μm.

Interestingly, at 14dpr, immature mice had a low abundance of cells matching the osteochondrogenic cluster, whereas mature mice exhibited a relatively increased proportion of osteochondrogenic cells (Figure 2b,c). These results were consistent with the histological findings in which the resected rib space of immature mice consisted mainly of newly formed and well differentiated osteochondrogenic/mineralized tissue by 14dpr, while the resection site in mature mice consisted of chondroblast‐like cells embedded in a chondroid matrix without any sign of bone formation (Figure 2e). scRNAseq data analysis revealed that in immature mice, cells expressing the key chondrogenic markers *Sox9* and *Col2a1* are localized to a single region in the osteochondrogenic cluster (#2) correlating with *Runx2*, which is crucial for osteogenesis (Figure 3a). However, in mature mice, multiple *Runx2*‐subpopulations exist separate from the *Sox9*‐ and *Col2a1*‐expressing cells which also overlap with other high density *Fn1* (fibronectin‐1) regions, associated with tissue repair and fibrosis (Figure 3b). Immunohistochemical (IHC) staining in the repair region of immature mice shows minimal fibronectin‐positive cells and several Runx2‐positive cells (Figure 3c), whereas in mature mice, most of the repair region included fibronectin‐positive cells and no Runx2‐positive cells were observed (Figure 3d). Together, these findings suggest that there are obvious differences in the composition of cells involved in the tissue repair of immature versus mature mice, and that may be playing a role in the regenerative outcomes.

**FIGURE 4:**
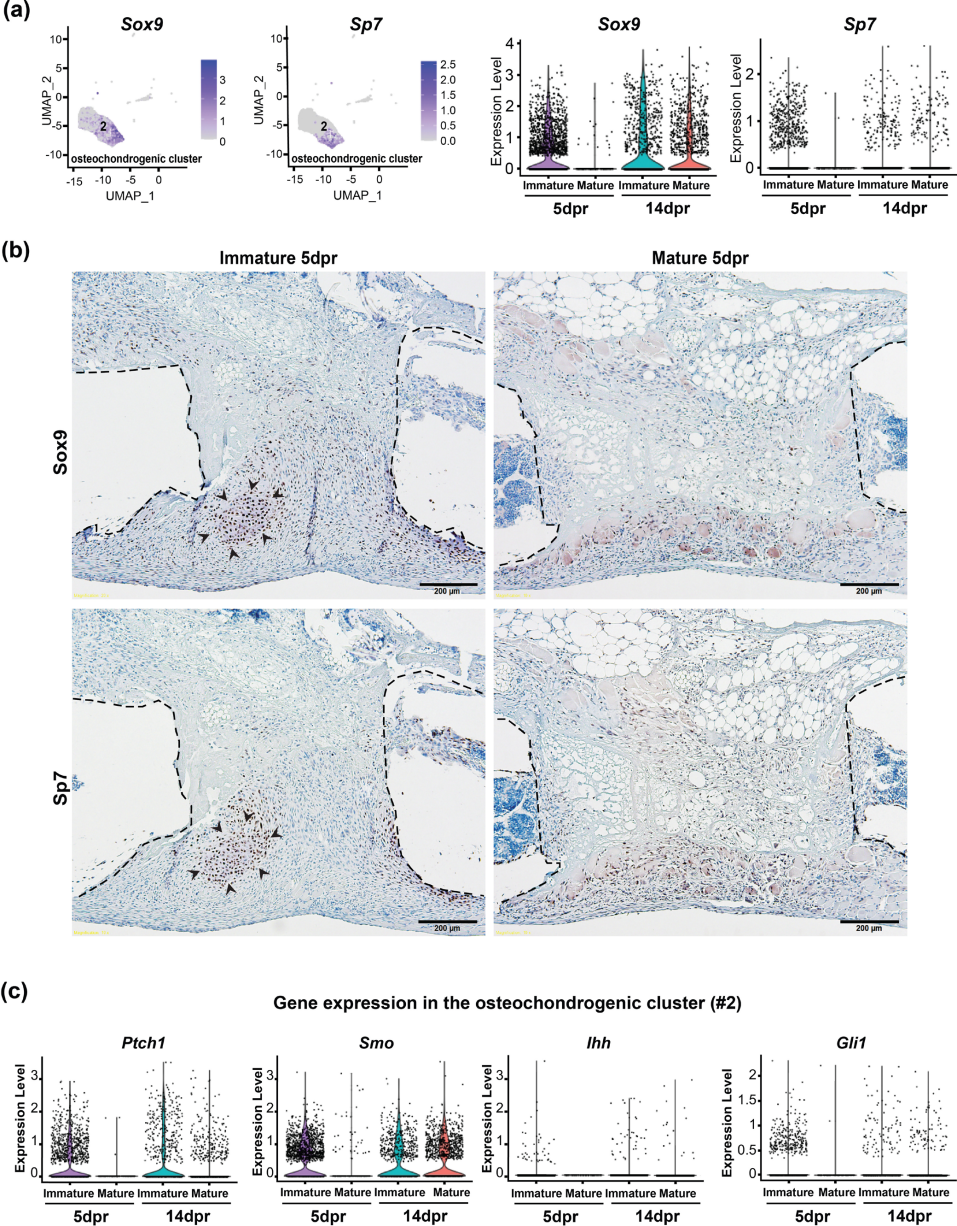
The spatial distribution and expression of SOX9 and SP7 are associated with skeletal maturity and age‐related outcomes of large‐scale rib defect repair. (a) UMAP representation (left) and normalized average relative expression of *Sox9* and *Sp7* (right) in the osteochondrogenic cluster (#2) identified by scRNAseq. Clusters were generated using a resolution of 0.1. All expression values are normalized and scaled averages. (b) Immunohistochemical representation of SOX9^+^ and SP7^+^ cells in the repair calluses of immature and mature mice at 5 dpr. Black arrowheads indicate the area of hypertrophic chondrocyte differentiation. The dashed black lines represent the ends of the ribs after resection surgery. Scale bars: 200 μm. (c) Violin plots depict the relative expression of key Hh pathway genes in the osteochondrogenic cluster of immature versus mature mice at 5 dpr and 14dpr. All expression values are normalized and scaled averages.

Previous studies using the same large‐scale injury mouse model show that *Sox9* ^+^ cells co‐express chondrocyte and osteoblast markers during the early stages of callus formation (Kuwahara et al. 2019; Serowoky et al. 2022). Here, using scRNAseq, we found that the relative expression levels of *Sox9* and *Sp7* in the osteochondrogenic cluster were reduced in mature mice when compared to immature mice at 5dpr (Figure 4a). These differences were no longer prominent at 14dpr, suggesting that *Sox9* and *Sp7* play an important role in the early stages that precede osteogenesis. Furthermore, IHC analysis results showed that SOX9^+^ and SP7^+^ cells were less abundant in the injury site of mature compared to immature mice at 2dpr (Figure S2). Most notably, we identified that immature mice exhibited an expression pattern of SOX9 and SP7 that was spatially different from mature mice at 5dpr. Strong nuclear expression of SOX9 and SP7 was predominantly in the areas of mesenchymal condensation with hypertrophic chondrocytes in the callus of immature mice, whereas the area of defective healing in mature mice showed sparse distribution of SOX9^+^ and SP7^+^ cells with scant cellularity overall (Figure 4b). There were no substantial differences in Ki‐67 distribution between the groups studied at 5dpr, suggesting that cell proliferation is not a major factor in the early stages of repair of large rib defects (Figure S3a).

Functional analyses indicate specific signaling requirements for bone healing, depending on the type of injury. The pro‐regenerative ability of the rib to repair large segments in mice occurs through a cartilage‐mediated process similar to endochondral ossification that depends on the activation of Hedgehog (Hh) signaling in *Sox9* ^+^ periosteal cells, but this pathway is not required for small‐scale fracture healing (Kuwahara et al. 2019; Serowoky et al. 2022). As expected, further analysis of the osteochondrogenic cluster in the current study showed that key genes of the Hh pathway are downregulated in mature mice at 5dpr (Figure 4c). Moreover, it was previously demonstrated that inhibition of the Hh signaling in *Sox9* ^+^ cells resulted in impaired large‐scale rib regeneration accompanied by reduced infiltration of other cell types, which possibly participate in osteochondral differentiation, into the callus (Serowoky et al. 2022). Thus, skeletal maturity and age‐related differences in the organization of distinct cell populations in bone calluses between groups may be related, but not solely linked, to the level of Hh signaling activation in *Sox9* ^+^ cells, since the absence of repair in mature mice shown here is significantly more severe than that previously reported in animals with deficient Hh signaling (Serowoky et al. 2022). Furthermore, we identified the *Smad4* (SMAD family member 4) gene as a potential driver of the different spatial distributions of SOX9^+^ and SP7^+^ cells between the two groups of mice studied. *Smad4* which encodes a core signaling mediator protein common to transforming growth factor‐β (TGF‐β) and bone morphogenetic protein (BMP) signaling pathways, is important for chondrocyte polarization and pre‐cartilaginous mesenchymal condensation (Lim et al. 2015; Whitaker et al. 2017), and was found significantly upregulated in *Sox9*‐expressing cells analyzed in the osteochondrogenic cluster (#2) of immature versus mature mice (Figure S3b, Table S3, and Data S1). This idea is supported by IHC analysis, which showed that nuclei staining of SMAD4 at 5dpr was striking in areas of hypertrophic chondrocyte differentiation in the repair callus of immature mice but not in the mature group (Figure S3c).

Together, our data suggest that the expression level of osteochondrogenic genes and the spatial tissue distribution of transcription factors important for chondrogenic and osteogenic differentiation in the early stages of repair of large rib defects are associated with bone regenerative outcomes.

### 2.4 Local and Systemic Immune Responses Are Altered During Initial Callus Formation in Mature Mice

As the early repair phase is already marked by differences in the osteochondrogenic phenotype, we additionally performed Ingenuity Pathway Analysis (IPA, QIAGEN, USA) of differentially expressed genes (DEGs) from immature versus mature mice to identify canonical signaling pathways potentially involved in this process. Cellular immune response and cellular stress were the only pathways significantly activated at 2 versus 0dpr (Figure S4; Data S2, S4). Accordingly, the acute inflammatory reaction that follows bone fracture is essential for initiating the repair process (El Khassawna et al. 2017; Loi et al. 2016; Rapp et al. 2016; Schlundt et al. 2018), while age‐related chronic inflammation has been closely associated with a decline in the regenerative potential of SSPCs (Ambrosi et al. 2021; Josephson et al. 2019; Loi et al. 2016; Mancinelli and Intini 2023). However, skeletal maturity and age‐related changes in the immune system have never been characterized during the repair of large‐scale rib defects. In this study, in addition to the osteochondrogenic cluster, scRNAseq analysis identified the presence of distinct cell populations differentially distributed between the two experimental groups and composed mainly of immune cells in the early and late stages of rib regeneration (Figure 2a–c). Indeed, histological analysis confirmed that endochondral bone regeneration in immature mice is preceded by a focal infiltration of mononuclear cells in the center of the soft callus at 2dpr and restricted to its periphery at 5dpr. In contrast, defective callus formation in mature mice exhibits fibrin‐rich granulation and few immune cells at 2dpr, which become more apparent in its center at 5dpr (Figure 2e). Consistently, IHC analysis showed that the spatial organization of CD45^+^ immune cells was distinct between groups and accompanied by differences in the distribution pattern of CD31^+^ blood vessels (Figure S5a,b).

**FIGURE 5:**
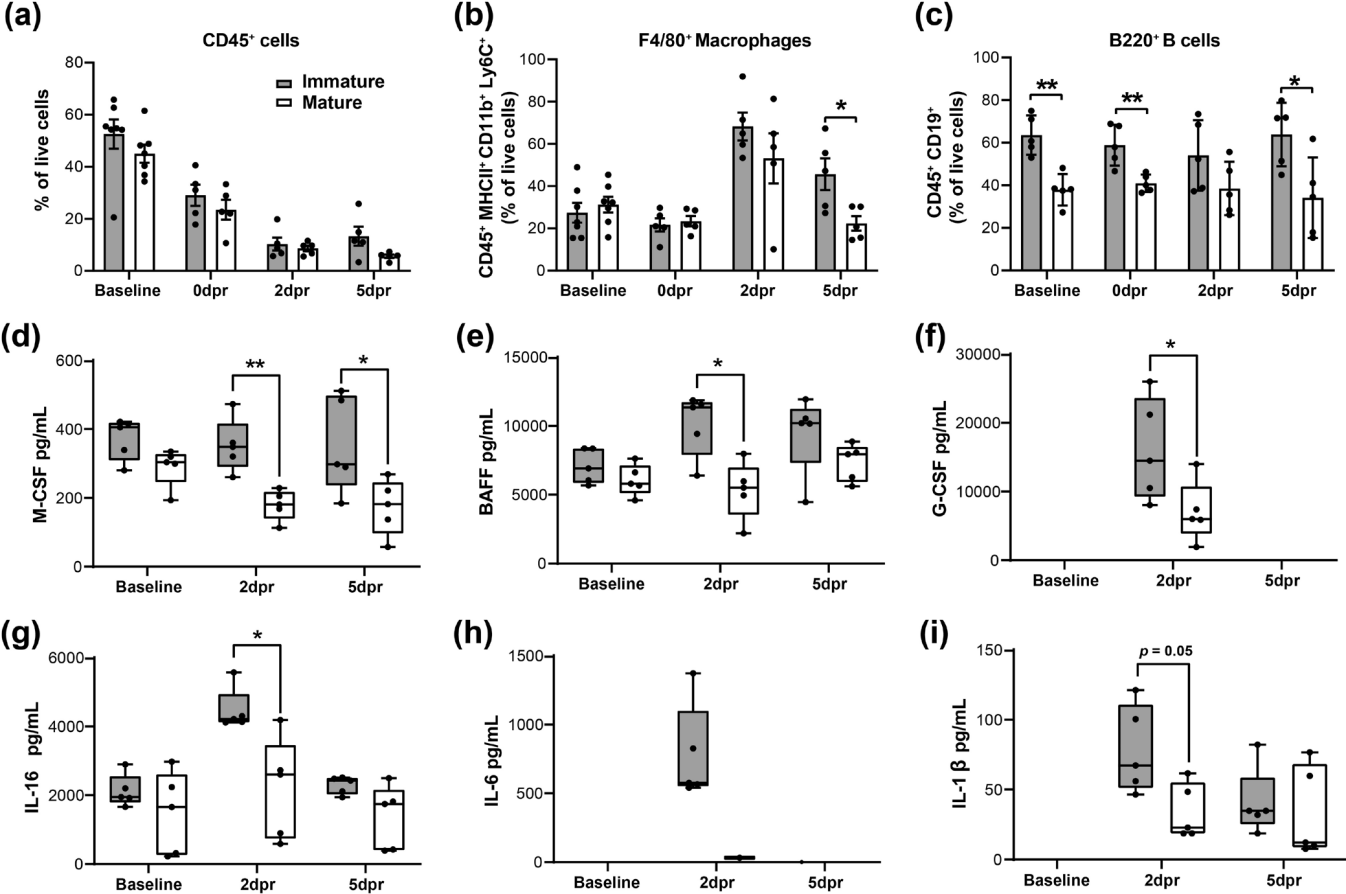
Skeletal maturity and age alter the local and systemic immune components during the early repair phase of large‐scale rib defects. Summary plots of flow cytometry data demonstrating the percentage of total CD45^+^ immune cells (a), F4/80^+^ macrophages (b), and CD19^+^B220^+^ B cells (c) in the repair calluses of immature and mature mice at baseline, 0, 2, and 5dpr (*p < 0.05, **p < 0.01; *n* = 5 mice or more/group). In all flow cytometric analyses, cells were pre‐gated on single, live cells, and specific immune cell subtypes were also pre‐gated in CD45^+^ cells (Figure S13). (d–i) Plasma concentrations of various cytokines measured by Luminex (pg/mL) in immature and mature mice at baseline, 2, and 5 dpr (*p < 0.05, **p < 0.01; *n* = 5 mice/group). An unpaired *t* test was used to find differences between immature and mature groups. Each dot represents a data point from an individual mouse, bars represent means ± standard error of mean (SEM), and boxes represent the minimum, median, and maximum values.

Although no significant differences were observed in the percentage of total CD45^+^ cells in the calluses during the early phase of repair using flow cytometric analysis (Figure 5a), F4/80^+^ macrophages peaked 2dpr in both groups and were significantly reduced in mature mice at 5dpr, with no differences in the expression of CD206, a cell‐surface protein abundantly present in anti‐inflammatory macrophages (Figures 5b and S6a,b). Interestingly, a baseline comparison already showed mature mice exhibiting a relative decrease in the percentage of CD19^+^B220^+^ B cells that remained similar from 0 to 5dpr (Figure 5c). Furthermore, within B cell subpopulations, while the percentage of CD19^+^B220^low^ B cell subtype was significantly reduced at 5dpr, CD19^+^B220^high^ B cells showed a clear tendency to increase at 2 and 5dpr in the repair calluses of mature mice (Figure S7a,b). On the other hand, unlike F4/80^+^ macrophages, immunostained B220^+^ B cells were less frequently observed at the resection site (Figure S7c). At baseline, the percentage of total CD3^+^ T cells did not change between groups, but the CD4^+^/CD8^+^ T cell ratio was significantly increased in mature mice (Figure S8a–d). In contrast, at 5dpr, the percentage of total CD3^+^ T cells was significantly higher in mature mice, while CD4^+^ and CD8^+^ T cell subpopulations exhibited similar frequencies. Finally, no significant differences were detected in the percentage of natural killer (NK) cells, NKT cells, dendritic cells, monocytes, and neutrophils (Figure S8e–i).

As distinct systemic immune responses have been correlated with positive or negative outcomes of bone repair after injury (Cheng et al. 2021), we next analyzed 31 secreted soluble factors (Table S4) present in plasma samples collected from the peripheral blood of immature and mature mice at baseline, 2 and 5dpr by using Luminex assays. Among them, we identified four circulating factors with significantly lower levels in mature mice at the early stages of bone repair: (1) macrophage colony‐stimulating factor (M‐CSF), relevant for the proliferation, activation, and maturation of monocytes/macrophages (Figure 5d); (2) B‐cell activating factor (BAFF), important for B cell maturation (Figure 5e); (3) granulocyte colony‐stimulating factor (G‐CSF), a key regulator of neutrophil production and differentiation (Figure 5f); and (4) interleukin (IL)‐16, a chemotactic factor for immune cells (Figure 5g). It is noteworthy that IL‐6, a cytokine relevant to inflammation and B cell maturation, was only detected at 2dpr and present in all immature mice, but only expressed at a low level in one mouse from the mature group (Figure 5h). Except for the tendency of the cytokine IL‐1β (produced by activated macrophages, monocytes, and dendritic cells) to be reduced in mature mice at 2dpr (Figure 5i), the other 25 plasma circulatory factors analyzed showed no significant difference between groups or did not exhibit detectable levels (Figure S9).

Together, our data show that altered infiltration of macrophages and lymphocytes during the early stages of callus formation coincides with decreased levels of circulatory pro‐inflammatory factors in mature mice, suggesting that skeletal maturity and age‐related local and systemic changes in immune components during the initial stages of repair are linked to impaired bone regeneration.

### 2.5 Interactions Between Immune and Osteochondrogenic Cells Could Potentially Influence Bone Regeneration

**FIGURE 6:**
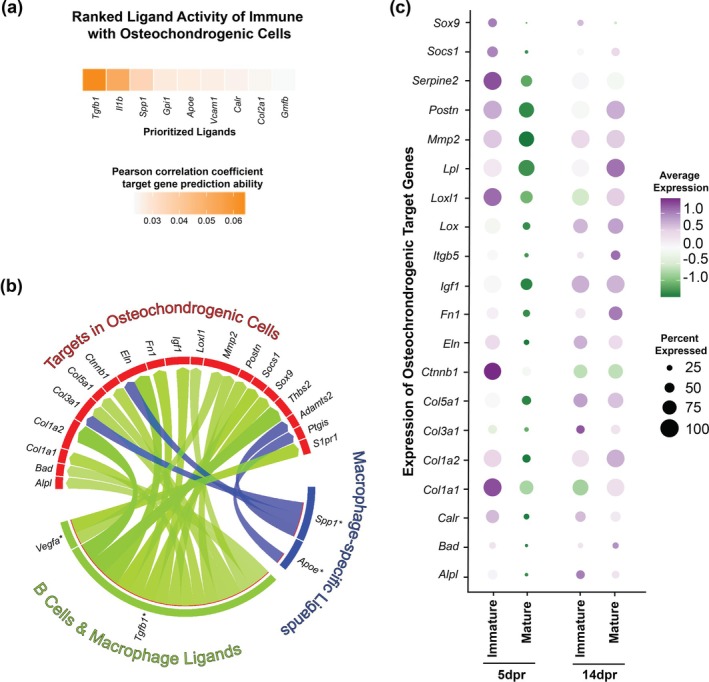
Immune cell‐derived ligands are potential upstream signals for osteochondrogenic differentiation. (a) Top ligand activity prediction in macrophages and B cells identified by NicheNet from scRNAseq data. Ranking of ligand activity analyses used the Pearson correlation coefficient. (b) Circos plot depicting links between ligands from macrophages and B cells and their predicted target genes in osteochondrogenic cells. (c) Dot plots highlight select top‐ranking predicted target genes in osteochondrogenic cells of immature and mature mice at 5 and 14dpr in the osteochondrogenic cluster (#2). Percentage of gene expression is represented by the area of a circle, and gene expression levels are color‐coded, ranging from purple (high expression levels) to green (low expression levels). Expression values are normalized and scaled averages. Together, our data suggest that age‐related factors compromise intercellular communication between immune and osteochondrogenic cells in response to injury in the early stages of repair, which likely impacts bone regenerative potential.

To test if immune cells could be potentially interacting with osteochondrogenic cells and influencing the expression of osteochondrogenic target genes, we set out to predict intercellular crosstalk using NicheNet analysis (https://github.com/saeyslab/nichenetr) from scRNAseq data based on ligand‐receptor pairs that are differentially expressed and active between cell clusters of interest. In addition to osteochondrogenic cells (cluster #2), macrophages (cluster #0) and B cells (cluster #4) were selected as distinct cell subgroups since the flow cytometric analysis showed that they represent the immune cell populations significantly decreased within the defective callus of mature mice and possibly involved in skeletal maturity and age‐related intercellular communications. We summarized these predicted cell–cell communications using a circos plot, where ligands expressed specifically in macrophages, or common to both macrophages and B cells, have the potential to interact with osteochondrogenic receptors (Figure S10a). *Tgfb1, Il1b, Spp1, Gpi1*, and *Apoe* were identified among the top five potential ligands expressed in macrophages and B cells capable of inducing transcriptional changes occurring in osteochondrogenic cells (Figure 6a). Furthermore, a decrease in the relative abundance of total *Ptprc* ^+^ immune cells, *Adgre1* ^+^ macrophages, or *Cd19* ^+^ B cells expressing the top predicted ligand *Tgfb1*, and osteochondrogenic cells encoding the corresponding TGF‐β receptors (e.g., *Tgfbr1/2*) was observed in mature mice (Figure S10b).

Interestingly, key regulators of osteogenic and chondrogenic differentiation pathways were among the top 20 predicted target genes in osteochondrogenic cells, potentially regulated by prioritized ligands expressed by macrophages and B cells (Figures 6b and S10c). Of note, the relative expression levels of all top‐ranking osteochondrogenic target genes, such as *Col1a1*, *Col1a2*, *Col3a1*, and *Sox9*, among others, were relatively reduced in the calluses from mature mice at 5dpr (Figure 6c).

Together, this cell–cell communication prediction analysis suggests that macrophages and B cells communicate with osteochondrogenic cells, potentially regulating their behavior.

### 2.6 Impaired Function of Macrophages and Lymphocytes Alters Proper Callus Formation

**FIGURE 7:**
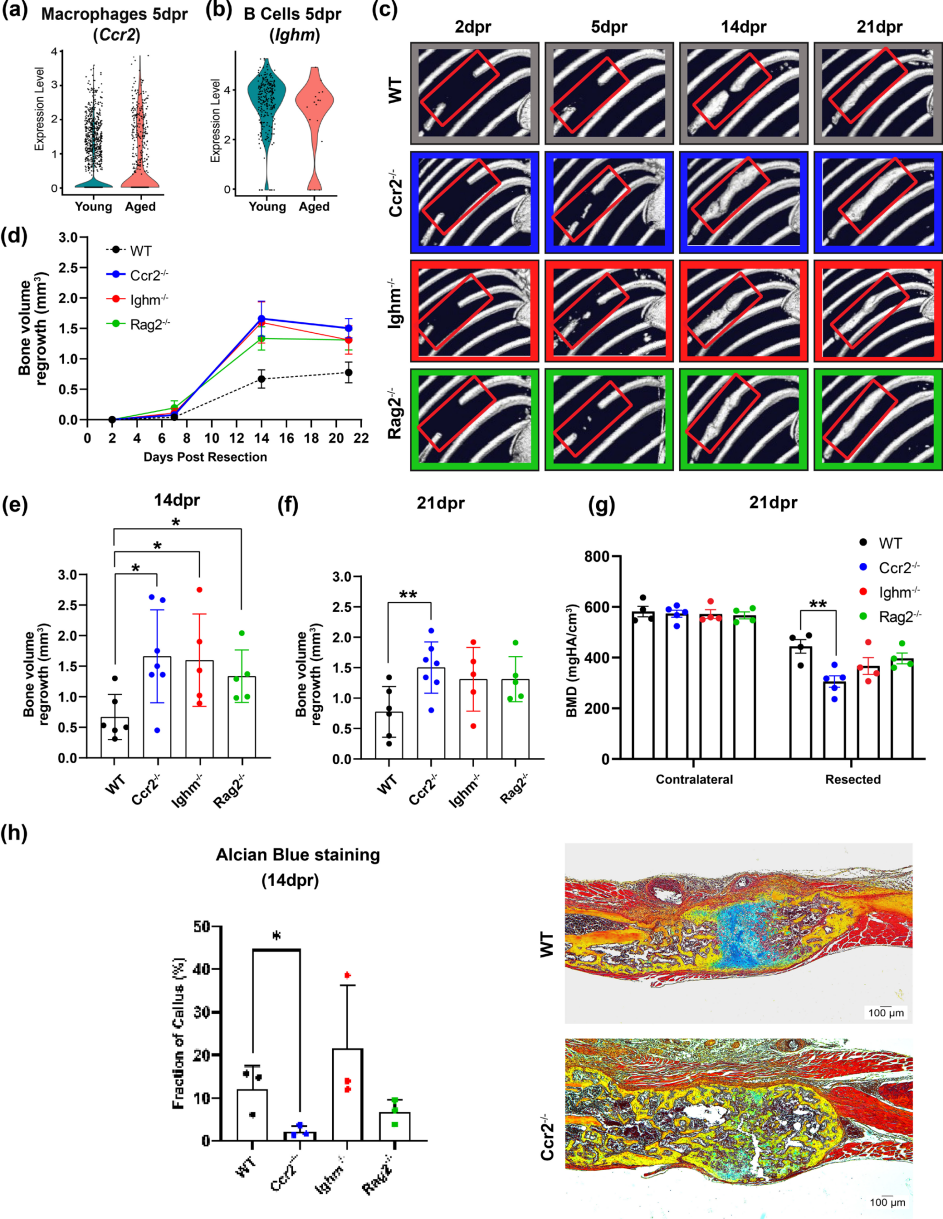
Defective immune cell function in immature mice impacts rib callus formation. Violin plots show the relative gene expression of *Ccr2* in (a) macrophages and *Ighm* in (b) B cells from immature and mature mice at 5dpr (*n* = 2 mice/group). Expression values obtained from scRNAseq analysis are normalized and scaled averages. (c) Representative live μCT images of large‐scale bone repair outcomes in WT, *Ccr2* ^−/−^, *Ighm* ^−/−^, and *Rag2* ^−/−^ immature mice at 2, 5, 14, and 21dpr. The red rectangles highlight areas of the ribs that were surgically resected and followed for any sign of bone regeneration from 2 to 35dpr in WT, *Ccr2* ^−/−^, *Ighm* ^−/−^, and *Rag2* ^−/−^ immature mice. (d) Line graph of the bone volume (mm^3^) for each mouse group over time. Bone volume regrowth (mm^3^) for each mouse group at 14dpr (e) and 21dpr (f). All bone volume measurements were obtained from live μCT data analyzed using AnalyzePro 14.0 software (*n* = 5–7 animals/group, unpaired Student's *t* test; *p < 0.05, **p < 0.01). (g) BMD (mgHA/cm^3^) of the rib calluses in each mouse group at 21dpr. All BMD measurements were obtained from ex vivo high‐resolution μCT data and analyzed using AnalyzePro 14.0 software (*n* = 4–5 animals/group, unpaired Student's *t* test; **p < 0.01). (h) Fraction (%) of cartilage/total callus area based on the quantification of Alcian blue positive staining in the rib calluses for each mouse group at 14dpr with representative histologic images of rib calluses from WT and *Ccr2* ^−/−^ mice shown (*n* = 3 animals/group, unpaired Student's *t* test; *p < 0.05). Scale bars: 100 μm; Movat–Pentachrome staining.

Owing to the relatively reduced frequency of macrophages and B cells in mature mice and the possibility that immune cell changes are induced by skeletal maturity and age, we reasoned that we would find changes in gene expression in these immune cell subtypes. Thus, we extended our scRNAseq analysis, which confirmed altered expression of the genes *Ccr2*, which encodes a receptor essential for the recruitment of macrophages, and *Ighm*, involved in the maturation of B cells (Figure 7a,b). In order to test whether altered immune cell function impacts rib regeneration, we used immature wild‐type (WT) and knockout (KO) mice lacking *Ccr2*, *Ighm*, or *Rag2* (a gene relevant for B and T cell maturation) of the same genetic background (C57BL/6). In vivo μCT analyses showed that all three KO mice had an increase in bone formation when compared to WT mice, with significantly larger calluses at 14dpr (Figure 7c–e) and *Ccr2* ^−/−^ mice also at 21dpr (Figure 7c,f). We additionally performed ex vivo high‐resolution μCT analyses and demonstrated that bone mineral density (BMD) was significantly lower only in *Ccr2* ^−/−^ at 21dpr (Figure 7g). Consistent with these data, histological analysis showed that *Ccr2* ^−/−^ mice had an impaired endochondral ossification process, as demonstrated by the significant decrease of cartilage intermediate formation at 14dpr based on the area of Alcian blue positive staining (Figure 7h).

Taken together, our findings suggest that although specific changes in macrophage or lymphocyte function in immature mice cannot recapitulate the loss of rib regenerative capacity in mature mice, they negatively affect the rate of regeneration and structural composition of the callus during the repair of large‐scale defects, suggesting that skeletal maturity and age‐related inflammation are closely related to dysregulated bone homeostasis.

### 2.7 Heterochronic Parabiosis Reverses the Impaired Regenerative Capacity of Mature Mice and Dysregulates Callus Formation in Immature Mice

**FIGURE 8:**
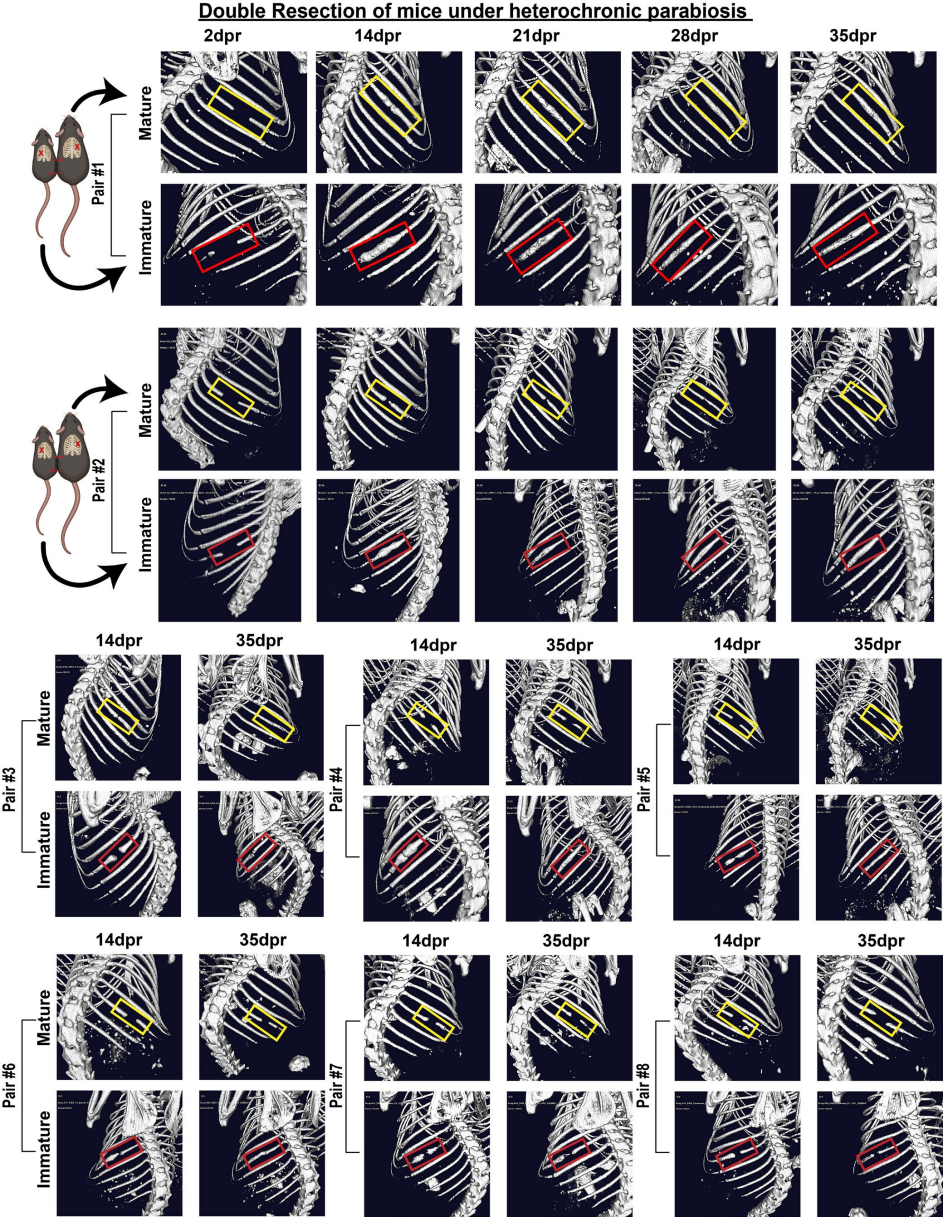
Double resection of mice under heterochronic parabiosis rescues large‐scale bone regeneration in mature mice. Representative live μCT images showing the expected callus formation in immature mice and the rescue of large‐scale bone regeneration in 3 out of 8 mature mice under heterochronic parabiosis in which both animals of the pair underwent rib resection. The double‐resected parabiotic pairs were scanned at 2, 14, 21, 28, and 35dpr. The red and yellow rectangles highlight areas of the ribs that were surgically resected and followed for any sign of bone regeneration from 2 to 35dpr in immature and mature mice, respectively. Cartoons were created with BioRender.com, showing illustrative images of relatively small and large mice representing immature and mature mice, respectively, under heterochronic parabiosis. The red “X” on the chest of both mice in each parabiont pair illustrated represents double rib resections.

It is crucial to examine whether tissue‐resident cells solely determine the capacity for regeneration or if circulatory factors also play a role during bone repair. This is particularly significant considering that the inflammatory reaction following rib resection is important for modulating the callus microenvironment during extensive bone repair. Additionally, it is noteworthy that immature and mature mice exhibit different levels of circulating inflammatory factors in response to bone injury. To this end, parabiosis surgeries were performed using parabiotic mouse pairs between ROSA^mT/mG^ fluorescent reporter mice, which constitutively express the tandem dimer Tomato (tdTomato) protein, and WT mice of the same background (C57BL/6). Parabiotic pairs that effectively shared the blood supply, confirmed by the presence of tdTomato^+^ cells in the circulation of both mice (Figure S11), were selected for a second surgical challenge. We first performed large‐scale rib resections in only one mouse from each heterochronic or isochronic pair (single resection). In contrast to the previous effects of young blood improving fracture healing in older mice (Baht et al. 2015; Vi et al. 2018), in vivo μCT analysis showed that, similarly to what was found in mature isochronic controls (*n* = 3) (Figure S12a), resected mature mice paired with unresected immature mice were unable to fully regenerate the rib, even after 35dpr (*n* = 3) (Figure S12b). This, however, might be because immature, unresected mice were not stimulated to produce factors that could promote bone regeneration. Then, we tested whether these results would be different when both immature and mature mice from each heterochronic pair underwent rib resection (double resection). Indeed, bone regeneration was prominent in 3 out of 8 mature mice under heterochronic parabiosis (Figure 8). Furthermore, we found that, in comparison to immature isochronic controls which showed incomplete rib regeneration at 14dpr (*n* = 3) (Figure S12c), immature mice under heterochronic parabiosis exhibit accelerated large callus formation, based on the complete connection between the rib ends at 14dpr (*n* = 3) (Figure S12d). Of note, large calluses from immature mice under heterochronic parabiosis resemble calluses from immature mice with impaired immune cell function (Figure 7c–f).

Taken together, these results show that skeletal maturity and age‐related changes in circulatory factors alter bone homeostasis, and that the systemic response capable of rescuing bone formation in mature mice is triggered exclusively after rib resection in immature mice.

## 3 Discussion

Although advancing age delays the healing of fractures (Clark et al. 2020), more pronounced reparative changes are observed after large‐scale bone injuries, as our study provides evidence that the capacity for bone regeneration declines abruptly with skeletal maturity and age until reaching a certain stage at which it is completely lost, suggesting that these different types of injury are not necessarily subject to the same regulatory mechanisms. Accordingly, it was previously demonstrated that activation of the Hh pathway signaling in periosteum derived *Sox9* ^+^ cells is crucial to promoting large‐scale rib regeneration but dispensable for small‐scale fracture healing in mice (Kuwahara et al. 2019; Serowoky et al. 2022). Consistent with this, we observed that large‐scale unhealed rib defects in mature mice are preceded by an initial soft callus with downregulation of *Sox9*, *Sp7*, and key components of the Hh pathway in osteochondrogenic cells. Of note, we also showed that the clustering of SOX9^+^ and SP7^+^ cells in corresponding areas of mesenchymal condensation with hypertrophic chondrocytes during the early stages of callus formation is a unique feature of immature mice. Despite the fact that SMAD4‐mediated signaling is critical for mesenchymal condensation and subsequent deposition of the cartilage matrix (Lim et al. 2015; Whitaker et al. 2017), it remains to be determined whether the disorganized distribution of SOX9^+^ and SP7^+^ cells in mature mice is solely associated with a skeletal maturity and age‐related decline in its expression or also influenced by other changes in the callus microenvironment. In this regard, an important feature observed at 14dpr is the abundant expression of fibronectin in unhealed bone defects of mature mice, suggesting that it may play a role in regulating the transition from inflammation to fibrosis instead of endochondral ossification during tissue repair.

It has been well described in fracture models that successful bone repair relies on an effective acute immune response (El Khassawna et al. 2017); however, there is also evidence that age‐associated chronic systemic inflammation can induce senescence and decrease the number and proliferation of SSPCs (Josephson et al. 2019; Mancinelli and Intini 2023), skewing them toward a pro‐inflammatory and pro‐osteoclastic stromal phenotype, which results in fragile bones with low regenerative capacity (Ambrosi et al. 2021). Furthermore, aged SSPCs promote imbalance in hematopoietic stem cells, polarizing them toward the production of myeloid cells (Ambrosi et al. 2021), which is considered a marker of hematopoietic aging with consequences for their functions (Eva and Carolina 2020). Here, we characterized skeletal maturity and age‐related changes in components of the immune system during the early stages of large‐scale bone repair and demonstrated that altered infiltration of macrophages and lymphocytes in the callus of mature mice coincides with decreased plasma levels of the pro‐inflammatory cytokines M‐CSF, BAFF, G‐CSF, IL‐16, and IL‐6. Based on these observations, we believe that tissue repair and regeneration may be influenced by both local and systemic immune responses following large‐scale bone injury. Accordingly, IL‐6 has been proven to be a regulatory factor in immune cell recruitment and endochondral ossification (Prystaz et al. 2018). However, the signals produced by immune cells that stimulate osteochondrogenic differentiation during the repair of large rib defects are still unknown. Our NicheNet analysis predicted enriched interactions between immune cells and osteochondrogenic cells primarily through TGF‐β signaling, suggesting a potential role of this pathway in regulating the expression of several transcription factors, including the identified target gene *Sox9*. In fact, multiple immune cell types express TGF‐β superfamily ligands and/or receptors that possess autocrine, paracrine, or even endocrine functional effects (Tamayo et al. 2018). Our findings are in line with a previous study that demonstrated TGF‐β signaling activation in primary human osteoblasts when exposed to conditioned media from monocytic‐ and macrophage‐like cells (Ehnert et al. 2018). In addition, TGF‐β has previously been shown to mediate the phosphorylation and stabilization of SOX9 protein in chondrocytes (Coricor and Serra 2016), whereas mature mice exhibit disrupted TGF‐β signaling and a subsequent reduction in cartilage repair (Blaney Davidson et al. 2005; van Caam et al. 2016). Although circulating levels of TGF‐β1 did not differ between the groups of mice studied (Figure S9), future functional studies in a spatial context are necessary to investigate whether intercellular communication between immune and osteochondrogenic cells through TGF‐β and/or other pro‐regenerative signaling pathways within the callus are affected by skeletal maturity and age and subsequently associated with a decline in large‐scale bone regeneration. A previous study employed spatial transcriptomics to demonstrate an age‐related decline in digit tip regeneration driven at least in part by a metabolic shift associated with increased angiogenic signaling and altered bone structure (Tower et al. 2022). Blood vessels play an important role in regulating tissue metabolism and cellular composition of local microenvironments. In poorly vascularized fracture sites, chondrogenesis is favored over osteogenesis as an adaptive response to local lipid deprivation regulated by SOX9 expression in SSPCs (van Gastel et al. 2020). The altered pattern of CD31+ blood vessel distribution combined with the reduced expression of *Sox9* in the early stage of callus formation of mature mice in the current study suggests that the relevance of angiogenesis in the rib regeneration outcomes should be explored in future studies involving large‐scale bone defects.

It is well known that age‐related dysregulation of immune‐related genes can lead to a chronic inflammatory environment, which is associated with delayed fracture repair (Clark et al. 2017, 2020; Josephson et al. 2019; Mancinelli and Intini 2023). Likewise, our study reveals skeletal maturity and age‐associated changes in the expression of genes important for macrophage migration and lymphocyte maturation, altering callus formation, suggesting that the inflammatory environment of the callus may influence SSPC behavior, playing a role in large‐scale rib defects. However, this study only examined bone repair outcomes in the absence of specific functions of selected immune cell subtypes that did not prevent bone formation in *Ccr2* ^−/−^, *Ighm* ^−/−^, and *Rag2* ^−/−^ immature mice, in contrast to what was observed in WT mature mice. Thus, further studies should focus on the causal relationships between different combinations of skeletal maturity and age‐related environmental factors, including immune dysregulation, and changes in the intercellular communication in the callus niche responsible for establishing differences in bone regenerative capacity. A previous study revealed that *Ccr2* deficiency reduces F4/80^+^ macrophage migration but does not affect intramembranous bone healing (Biguetti et al. 2018), differing from results reported in the formation of periosteal calluses, as found in ribs, which occurs through endochondral ossification and has different mechanisms of cellular differentiation (Galea et al. 2021). Our findings indicate that *Ccr2* deficiency has a detrimental impact on endochondral ossification and BMD. This is consistent with previous studies demonstrating that macrophage depletion inhibits osteoblast differentiation (Chang et al. 2008) and decreases mineralization of fracture calluses (Wan et al. 2020). Furthermore, macrophage properties appear to be altered with age, as callus formation in young mice is abolished when macrophages are depleted at the time of fracture surgery (Raggatt et al. 2014), whereas inhibition of macrophage infiltration in aged skeletally mature mice improves the results of fracture healing (Clark et al. 2020). Regarding lymphocytes, it has been shown that fracture healing is accelerated in the absence of aged lymphocytes, but the elasticity and function of the repaired bone are lower (El Khassawna et al. 2017; Toben et al. 2011). Moreover, B‐cell exosomes were demonstrated to modulate osteoblast differentiation in vitro and bone formation in vivo (Zhang et al. 2021). Together, our data provide evidence that immune cells are major contributors to the pace and quality of repair of large bone defects and suggest that modulation of the immune response may optimize bone regeneration. Additionally, we believe that the aged microenvironment, including adipocytes and other cell types, may also play a role during tissue repair. Indeed, the literature shows that aging promotes the expansion of the adipocytic lineage, as previously demonstrated in bone marrow, and that the increased frequency of this population is negatively correlated with the potential for bone regeneration (Ambrosi et al. 2017). Future studies will help determine whether any specific skeletal maturity and age‐related differentiation pathways are present which promote or prevent the presence of specific populations supporting regeneration. Together, these data suggest that immune cells play a crucial role in regulating the function of SSPCs and the rate of endochondral ossification to generate quality bone.

Controversial data has pointed to heterochronic blood exchange as either promoting or failing to rejuvenate aged fracture healing (Ambrosi et al. 2021; Baht et al. 2015; Vi et al. 2018; Zhang et al. 2022). Herein, we demonstrate that impaired bone repair is not reversed in mature mice when their circulation is shared with unresected immature mice. However, it is noteworthy that this study provided evidence that, under heterochronic parabiosis in which both animals underwent rib resection, a systemic pro‐regenerative response is exclusively triggered in immature mice and is capable of rescuing bone regeneration in mature mice otherwise unable to regenerate spontaneously. Although age‐matched mice have been consistently used and purchased from the same source, we observed variable results across biological replicates in the heterochronic parabiosis experiments, specifically when immature and mature mice underwent rib resection (only 3 out of 8 mature mice displayed improved rib regeneration). Thus, we speculate that most likely, individual immature mice respond to bone injury with different levels of circulating pro‐regenerative factors and/or mature mice exhibit a distinct number of physiologically responsive SSPCs relative to senescent ones as they age. On the other hand, we consistently observed an accelerated formation of large calluses in immature mice when exposed to the blood of mature mice, previously characterized by relatively low levels of pro‐inflammatory circulatory factors. This accelerated pattern of bone formation resembles that of *Ccr2* ^−/−^, *Ighm* ^−/−^, or *Rag2* ^−/−^ immunodeficient mice when compared to WT, implying the involvement of a dysregulated immune response in both scenarios. Together, these data suggest that age alters the composition of immune cells and circulatory factors which have a subsequent effect on bone regeneration; however, the systemic regenerative response is exclusively generated in immature mice and depends entirely upon surgical procedures. Previous studies in murine models have demonstrated that SSPCs do not migrate systemically (Chan et al. 2015; Murphy et al. 2020), suggesting that systemic pro‐regenerative factors (i.e., cells and/or soluble molecules of an inflammatory or other nature) from immature mice may be capable of reprogramming tissue‐resident cells (including local SSPCs) in mature mice to support bone regeneration. Therefore, further research is needed to identify and define the combination of systemic factors that are necessary for reversing impaired regeneration of large bone defects.

## 4 Conclusion

Taken together, these results suggest that tissue‐resident immune cells and circulatory factors are affected by skeletal maturity and age and play a central role in bone regeneration. Appropriate identification of pro‐regenerative factors will be useful in the treatment of patients with extensive bone injuries.

### 4.1 Limitations

This study is limited by the fact that only female mice were used in this study, as is generally the case in parabiosis experiments due to their less aggressive behavior, and for comparative purposes between analyses of individual mice and those in parabiosis. Therefore, it is still an open question whether the changes in cellular and molecular characteristics related to skeletal maturity and age decline that we observe in females are also present in a similar way in males, or whether these characteristics are sex specific. In addition, the spatial resolution of in vivo μCT scans for animals under parabiosis was sacrificed to allow a lower radiation dose, which prevented quantitative data generation. Future studies that include additional identification and functional validation of pro‐regenerative circulatory factors, as well as identification of the spatial localization of the signals that drive rib regeneration, may provide some new perspectives on the mechanism underlying the ribs' apparent skeletal maturity and age‐dependent large‐scale bone regeneration capacity.

## 5 Materials and Methods

### 5.1 Patients and Specimens

All experiments involving human subjects were approved by the University of California, San Francisco (UCSF) Institutional Review Board (CHR #H42089‐22594). This prospective cohort study involved patients with a craniofacial bone defect or congenital deficiency that required a costochondral rib graft who were evaluated in the UCSF Oral & Maxillofacial Surgery Clinic between 2004 and 2008. This protocol included patient clinical data collection at the time of surgery and postoperative chest CTs to follow bone regeneration. Each patient underwent immediate postoperative volumetric CT (mm^3^). Two subsequent CTs were obtained during the routine 3‐ and 6‐month postoperative visits. Details of the CT imaging and analysis of rib regeneration are reported in the Supporting Information files. Patient data from a 57‐year‐old woman were omitted to avoid any bias due to potential physical/metabolic changes related to hormonal interference, other than skeletal maturity and age.

### 5.2 CT Imaging and Analysis of Rib Regeneration in Humans

CT imaging was performed with the 16‐row multi‐slice CT scanner (Lightspeed, GE, Milwaukee, Wisconsin). The peak voltage (kVp) and electric current (mA) values used were based on the weight of the patient. 3.75 mm was determined as the image slice thickness and reconstructed to a thickness of 1.25 mm. CT image acquisition was restricted to the site of resection marked with a radio‐opaque marker, thus limiting radiation exposure to the patient. A standard bone mineral density calibration phantom (MINDWAYS, Austin, Texas) was included in the field of view for standardizing the analysis and quantification of density. A standardized window width of 1500 Hounsfield Units (HU) and a level of 500 HU were selected to optimize bone and callus visualization and to establish a clear, reproducible distinction from the surrounding soft tissue. To quantify the bone regeneration, the site of resection was identified on the baseline image set. Using the digital ruler feature of the image analysis software, the distance from the surgically resected edge was measured to a reproducible bone landmark (den Boer et al. 1998; Roth et al. 1997).

### 5.3 Animals

All the animal experiments were approved by the NIH/NIDCR Animal Care and Use Committee (Protocol numbers: #18‐869, #21‐1081, #20‐1034, and #23‐1130) and conducted in accordance with ARRIVE guidelines (https://arriveguidelines.org). For phenotype characterization, female C75BL/6 mice (Strain: #027), immature (≤ 2‐months), human‐equivalent ages of 12–16 years, and mature (≥ 10‐months), human‐equivalent ages of 38–47 years, were obtained from Charles River Laboratories and selected to undergo rib resection surgery. To assess the global role of specific immune cells in bone structure and regeneration processes, we used female WT, *Ccr2* ^−/−^ (Strain: #004999), *Ighm* ^−/−^ (Strain: #002288), or *Rag2* ^−/−^ (#008449) mice of the same genetic background (C75BL/6) obtained from the Jackson Laboratory. For parabiosis experiments, female WT mice (Strain: #000664) and ROSA^mT/mG^ mice (B6.129(Cg)‐Gt(ROSA)26Sortm4(ACTB‐tdTomato, ‐EGFP)Luo/J‐Strain: #007676) of the same genetic background (C75BL/6) were originally from the Jackson Laboratory. The ages ≤ 2‐months for immature mice and 8‐months for mature mice were used in order to limit the size difference between the two animals under heterochronic parabiosis.

### 5.4 Murine Rib Resection Surgery

Rib resections were performed as previously described (Kuwahara et al. 2019; Tripuraneni et al. 2015; Yamamoto de Almeida et al. 2024).

### 5.5 In Vivo and Ex Vivo High‐Resolution μCT Imaging and Analysis of Rib Regeneration in Mice

For the acquisition of in vivo μCT scans, mice were anesthetized with 2% isoflurane and scanned on a Quantum GX microCT (PerkinElmer Inc. Waltham, MA). The X‐ray source was set to a current of 88 μA and a voltage of 90 kV. The field of view (FOV) was set to 36 mm × 36 mm, and voxel size was 72 μm for a single animal, and the FOV was set to 72 mm × 72 mm, and voxel size was 144 μm for mice under parabiosis. For the acquisition of ex vivo high‐resolution μCT scans, mice were euthanized, ribs were dissected and fixed for 48 h at 4°C in paraformaldehyde (PFA), 4% in PBS, and then stored in 70% ethanol at 4°C. 3D reconstruction images of the ribs were acquired using μCT 50 (Scanco Medical AG, Bassersdorf, Switzerland) at 70 kVp X‐ray source voltage, 85 μA of intensity/beam current, power at 6 W and integration time at 900 ms. The image resolution was 10 μm. The 3D rib images were rendered, and the BMD of cortical bone and intertrabecular tissue was measured using AnalyzePro 14.0 software (AnalyzeDirect, Overland Park, KS, USA). All bone volume (mm^3^) measurements were obtained from in vivo μCT image analysis. After uploading the DICOM files to AnalyzePro software, the regenerated rib (5, 14, and 21dpr) was first segmented from its adjacent bone ends (vertebral column and costal cartilage). Then, the total bone volume value of the regenerated rib (5, 14, or 21dpr) was normalized by subtracting it from the total bone volume value of the respective contralateral rib. Finally, the resulting value obtained from the initial subtraction was then again subtracted from the total volume measurement obtained after rib resection (2dpr) to compensate for sample variations.

### 5.6 Histology

Ribs collected at 2, 5, and 14dpr were placed in PFA 4% in PBS for 48 h. Then, the samples were decalcified in a 10% ethylenediaminetetraacetic acid (EDTA) solution and processed at the NIDCR Histology Core, and formalin‐fixed paraffin‐embedded (FFPE) blocks were created. Serial cuts (6 μm) were made by microtome and transferred to glass slides. Select slides were stained using hematoxylin and eosin (H&E) and Movat‐Pentachrome (#ab245884, Abcam, USA). Cartilage was quantified based on the area of Alcian blue staining using Image J software. All images were taken using an Olympus BX51 microscope, an Olympus DP72 digital camera, and cellSens software.

### 5.7 Whole‐Mount Staining

Mice were euthanized at 14dpr, and the rib cage was placed in two changes of 95% ethanol overnight at room temperature, placed in 100% acetone for 2 days, incubated in Alcian blue for 1–3 days, and detained in two changes of 70% ethanol. Then samples were washed with 95% ethanol overnight and then placed in 1% potassium hydroxide (KOH) overnight. They were then stained in 1% KOH Alizarin Red for 2–5 days and then transferred to the 1% KOH solution. For storage and imaging, specimens were stored individually in 100% glycerol. Images were taken using a micro dissecting scope (Zeiss Stemi 508 Axiocam 4C 5S).

### 5.8 IHC

Sections (5 μm) were deparaffinized, rehydrated, and incubated with the antibodies listed in Table S5. Antibody detection was performed with Vectastain Elite ABC Kits (Vector Labs, USA) according to the manufacturer's protocol.

### 5.9 Flow Cytometry

Tissues were harvested at baseline, 0, 2, and 5 dpr, minced into ~1 mm sizes, and enzymatically digested at 37°C using collagenase type XI (0.05%) and dispase (0.06 units/mL) for 60 min and 40 min, respectively. Subsequently, single cell suspensions were subjected to flow cytometric analysis. First, cells were pre‐incubated for 10 min with purified anti‐mouse CD16/32 (clone 93, #101320, BioLegend) to block unspecific antibody binding and for 30 min with Brilliant Stain Buffer (#566349, BD Biosciences) to prevent artifacts from BD Horizon Brilliant fluorescent dye interactions. Then, *Blue Fixable Live*/*Dead cell* dye (#L23105, Invitrogen) was added in a 1:200 dilution to 1 million cells for 15 min to exclude dead cells from analysis, before staining with the appropriate antibodies for 40 min. The antibodies are listed in Table S6. Samples were acquired on a Cytek Aurora Spectral Analyzer with five lasers and analyzed with SpectroFlo software. The gating and cell identification strategies used to define all immune cells are shown in Figure S13.

### 5.10 Parabiosis

Our parabiosis surgical procedures were adapted from a previously published protocol (Kamran et al. 2013). Isochronic (immature C75BL/6, immature ROSA^mT/mG^, or mature C75BL/6, mature ROSA^mT/mG^) and heterochronic (mature C75BL/6, immature ROSA^mT/mG^, or mature ROSA^mT/mG^, immature C75BL/6) pairs were established 2 weeks before rib resection. At 10–14 days post‐parabiosis, flow cytometric analysis was conducted to determine shared circulation in parabiotic mouse pairs by assessing the relative percentage of live TdTomato^+^ cells in peripheral blood. Rib resection surgeries were performed on only one mouse from each pair (under isochronic or heterochronic conditions) or on both parabionts (only under heterochronic conditions) and animals were monitored for bone regeneration using μCT scans at 2, 14, 21, 28, and 35dpr.

### 5.11 Luminex Multiplex Array

Plasma isolates collected at baseline, 2, and 5dpr were analyzed for soluble target analytes using the Mouse XL Cytokine Luminex Performance Premixed Kit (#FCSTM20, R&D Systems, USA) and the TGF‐beta 1 Magnetic Luminex Performance Assay (#LTGM00, R&D Systems, USA). The assays were read in a Bio‐plex 200 system using Bio‐Plex Manager software 6.1. Analyses were performed according to the manufacturer's instructions, and the analyte concentration level was measured using a standard curve for each specific target analyte (Table S4).

### 5.12 RNA‐Seq Preparation and Data Analysis

RNA samples were extracted using Tripure (Sigma, USA) and Pure Link RNA Minikit (Thermo Fisher, USA), evaluated for concentration and quality using an Agilent Bioanalyzer, and subsequently sent to Genewiz (South Plainfield, NJ, USA) for library construction and sequencing.

Paired‐end sequencing data with 150 bp per read generated by Genewiz were subjected to the following analyses: (1) mapping against the mouse GRCm38 reference genome and measuring for expression levels (count values) of each gene with STAR 2.5.2a; (2) comparison across samples for significant differential expression with DESeq2 (adjusted *p* < 0.05); (3) plotting of between‐sample fold change values per gene using R (https://www.r‐project.org).

### 5.13 scRNAseq Preparation and Data Analysis

Repair calluses were dissected from immature and mature mice at 5 and 14dpr, minced into ~1 mm sizes, and enzymatically digested at 37°C using collagenase type XI (0.05%) and dispase (0.06 units/mL) for 60 min and 40 min, respectively. Subsequently, single cell suspensions of each group were pooled (*n* = 2) and loaded onto a chromium chip (10X Genomics) followed by encapsulation in a lipid droplet (Single Cell 3′ kit, 10X Genomics) to generate cDNA and a library according to the manufacturer's protocol. cDNA libraries were sequenced using Illumina Nextseq 500. scRNAseq reads were processed with Cell Ranger v2.1, which demultiplexed cells from different samples and quantified transcript counts per putative cell. Clustering was done using the Louvain method on quality‐filtered cells, with the “FindClusters” function using the first 20 PCs and a resolution of 0.1. Cells that expressed fewer than 200 genes, more than 5000 genes, and those with more than 10% mitochondrial gene expression were excluded. Gene expression matrices were transformed for better interpretability using the Seurat function “NormalizeData”. We used UMAP dimensional reduction to visualize clusters and the Wilcoxon Rank Sum test to identify differentially expressed genes. Density plots of genes were generated using the Nebulosa R package (Alquicira‐Hernandez and Powell 2021) to visualize data from single cells based on kernel density estimation. It aims to recover the signal from dropped‐out features by incorporating the similarity between cells, allowing a “convolution” of the cell features.

### 5.14 NicheNet Analysis

NicheNet interaction predictions were generated using expression data from scRNAseq and the vignette described at https://github.com/saeyslab/nichenetr. Osteochondrogenic cells (cluster #2) were set as “receiver” and B cells (cluster # 4) and macrophages (cluster #0) as “sender” populations. Circos plots of the top ligand‐receptor interactions were generated to visualize links between ligands on B cells and macrophages and receptors and targets on osteochondrogenic cells. The scoring of the predicted targets was based on a Pearson correlation coefficient as described in the NicheNet vignette. Seurat and ggplot2 were used for data visualization.

### 5.15 Statistical Analysis

For human data analysis, means ± standard deviation (SD) was compared between young and older patients using unpaired *t*‐tests with a confidence level of 95% and *p* < 0.05 considered to be statistically significant. Correlations were examined using the Pearson *r*. Stepwise multiple linear regressions of bone volume or density on age, gender, and time were performed with alpha = 0.05 to remain in the models. Volumetric measurements were calculated independently three times by the same individual. SAS statistical software (Cary, NC) was used for all statistical analyses. For animal data analysis, means ± SEM were used to express the number of observations. Student's *t* test was used for all comparisons in which there were two groups; one‐way analysis of variance (ANOVA) followed by Dunn–Sidak tests was applied for analyses in which there were two or more comparisons being made. Error bars in the figures represent SEMs. Statistical analysis was performed using GraphPad Prism 9, version 9.0.0. *p* < 0.05 was considered statistically significant. All experiments were performed on ≥ 3 individual biological samples; sample numbers are provided in Figure legends.

## Author Contributions

Conceptualization and design of the study: L.Y.A., O.D., J.S.L. Data curation: L.Y.A., C.D., A.S.H., K.M.M., D.M., NIDCD/NIDCR G.C.B.C., N.C., P.J., X.G., O.D., J.S.L. Formal analysis: L.Y.A., C.D., K.M.M., D.M., T.F., T.M.L., J.M.L., N.C., P.J., X.G., W.Y., O.D. Investigation: L.Y.A., C.D., K.M.M., D.M., T.F., T.M.L., J.M.L., N.C., P.J., X.G., W.Y., O.D. Methodology: L.Y.A., C.D., K.M.M., D.M., T.F., T.M.L., J.M.L., N.C., P.J., X.G., W.Y., O.D. Project administration: L.Y.A., A.S.H., N.C., O.D., J.S.L. Visualization: L.Y.A., A.S.H. Study supervision: O.D., J.S.L. Writing – original draft: L.Y.A. Writing – review and editing: L.Y.A., A.S.H., X.G., F.V.M., O.D., J.S.L. All the authors have read and approved the final manuscript.

## Conflicts of Interest

The authors declare no conflicts of interest.

## Supporting information


Data S1.

Data S2.

Data S3.

Data S4.

## Data Availability

All data presented in this manuscript are available within the paper and its Supporting Information files or deposited in NCBI GEO database (accession number for bulk RNAseq data: GSE250199/secure token: yxulwyksjjezvex; accession number for scRNAseq data: GSE250200/secure token: ctilsgcwpdsfnip).
