# Supplementary material for: Skeletal Maturity and Age‐Related Changes in Immune Cells and Circulatory Factors Impair Large‐Scale Bone Regeneration
Source: Aging Cell. 2025 Jul 21;24(10):e70177. doi: 10.1111/acel.70177 (PMC12507423; doi:10.1111/acel.70177)
Supplement: Supplementary file 1 — Data S1. [file ACEL-24-e70177-s003.docx]

**Supplementary Information**

**Skeletal maturity and age-related changes in immune cells and circulatory factors impair large-scale bone regeneration**

Luciana Yamamoto de Almeida^1^*, Catharine Dietrich^1^, Ashleigh Hanner^1^, Katelyn M. McTighe^1^, Daniel Martin^2^, NIDCD/NIDCR Genomics and Computational Biology Core^2^, Todd Fairbanks^3^, Thomas M. Link^3^, John M. Le^1^, Natasha Curry^1^, Priyam Jani^1^, Xin Gao^1^, Wenli Yu^3^, Francesca V. Mariani^4^, Olivier Duverger^1^, Janice S. Lee^1^*

Affiliations:

^1^Craniofacial Anomalies and Regeneration Section, National Institute of Dental and Craniofacial Research (NIDCR), National Institutes of Health; Bethesda, MD, 20892, USA.

^2^Genomics and Computational Biology Core, National Institute on Deafness and Other Communication Disorders (NIDCD)/NIDCR; Bethesda, MD, 20892, USA

^3^Department of Oral & Maxillofacial Surgery, University of California San Francisco, San Francisco, CA, 94143, USA

^4^Department of Stem Cell Biology and Regenerative Medicine, Keck School of Medicine, University of Southern California, 1425 San Pablo Street, Los Angeles, CA, 90089, USA

*Corresponding Authors:

Janice S. Lee, DDS, MD, FACS. Craniofacial Anomalies and Regeneration Section, National Institute of Dental and Craniofacial Research, National Institutes of Health, 10 Center Drive, Building 10, Room 5-2531, Bethesda, MD 20852

Email: janice.lee@nih.gov

Phone (Office): (301) 827-1647

Luciana Yamamoto de Almeida, DDS, PhD. Craniofacial Anomalies and Regeneration Section, National Institute of Dental and Craniofacial Research, National Institutes of Health, 30 Convent Drive, Building 30, Room 202, Bethesda, MD 20892

E-mail: yamamotodealmln@nih.gov

Phone (Office): (301) 827-5111

**Supplementary Materials and Methods**

**Pathway analysis**

Lists of DEGs with a −log(P-value) > 2 threshold for each comparison were analyzed using IPA^®^ (QIAGEN, USA). Top biological processes or pathways were associated with each network via Fisher’s exact test and enrichment scores (z-score). Adjusted P < 0.05 and the z-score (2.0 ≤ z ≥ 2.0) were considered significant.

**Functional enrichment analysis**

Enrichment analyses of the significant DEGs (log2 fold change > 0.5, adjusted P < 0.05) in repair callus at 0, 2, and 5dpr from bulk-RNAseq data were imported into Enrichr (<http://amp.pharm.mssm.edu/Enrichr/>) for determining biological processes and transcription factors that could explain the differential capacity of bone regeneration between immature and aged mice. We used gene set libraries as follows: GO Biological Process 2021, TRRUST Trancription Factors 2019, and TRANSFAC and JASPAR PWMs. The visualization of transcription factors hub networks and the calculation of P-value, odds ratio (OR), and combined scores were constructed by Enrichr. The results were analyzed by December 8, 2022.

**Figure S1** Bulk RNA sequencing reveals skeletal maturity and age-related changes in extracellular matrix organization and expression of osteogenic factors during the early stages of large-scale rib defect repair.

**Figure S2** The expression of SOX9 and SP7 are associated with skeletal maturity and age-related outcomes of large-scale rib defect repair.

**Figure S3** Assessment of Ki-67 and SMAD4 expression during the early-stage repair of large-scale rib defects.

**Figure S4** Bubble plot of differentially expressed genes between the calluses of immature and mature mice after rib resection.

**Figure S5** Differential spatial distribution of immune and endothelial cells in the callus of immature and mature mice.

**Figure S6** Skeletal maturity and age-related features of macrophage callus infiltration.

**Figure S7** skeletal maturity and age alter the infiltration of B cells during the repair of large-scale rib defects.

**Figure S8** Flow cytometric analysis of immune cells in rib calluses.

**Figure S9** Plasma levels of circulatory factors before and after rib resection.

**Figure S10** Crosstalk between immune and osteochondrogenic cells may play an important role during rib regeneration.

**Figure S11** Flow cytometric analysis of peripheral blood from mice before and after parabiosis.

**Figure S12** Live µCT analysis of rib repair outcomes in single-resected pairs of mice under isochronic and heterochonic parabiosis.

**Figure S13** Gating strategies for flow cytometry data analysis.

**Table S1** Cluster defining genes.

**Table S2** Enrichment analysis of upregulated genes from *Sox9*-expressing cells.

**Table S3** List of antibodies used for immunohistochemistry.

**Table S4** List of antibodies used for immunohistochemistry.

**Table S5** List of antibodies used for flow cytometry assays.

**
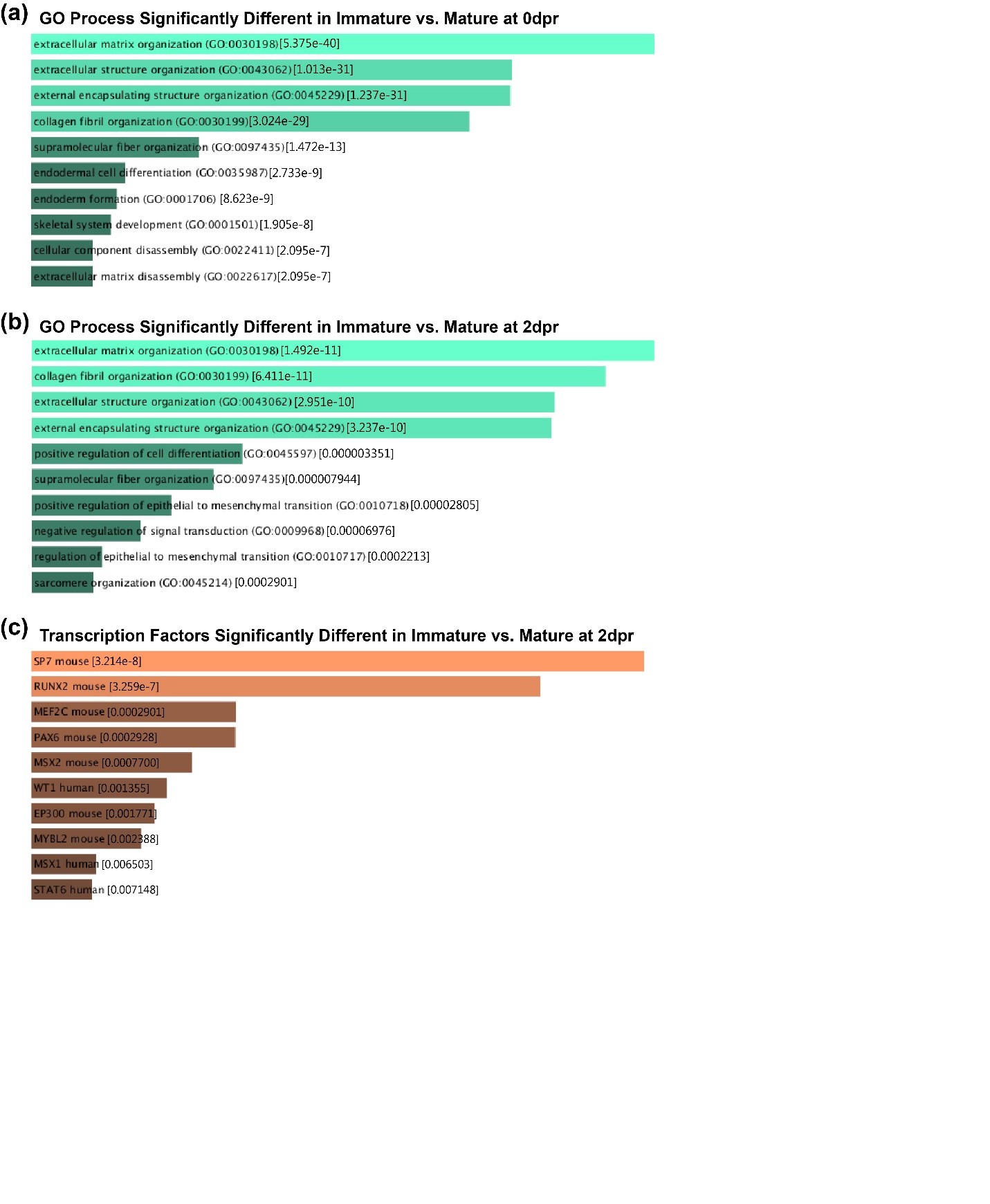
Figure S1** Bulk RNA sequencing reveals age-related changes in extracellular matrix organization and expression of osteogenic factors during the early stages of large-scale rib defect repair. The top 10 upregulated GO biological processes at 0dpr (**a**) and 2dpr (**b**) are ranked by the P-value associated with differentially expressed genes between mature and immature mice obtained by bulk RNA-seq and using the Enrichr analysis tool (http://amp.pharm.mssm.edu/Enrichr/). (**c**) Top 10 enriched transcription factor terms of DEGs between groups at 2dpr ranked by P-value using the TRRUST Transcription Factors 2019 database.

**
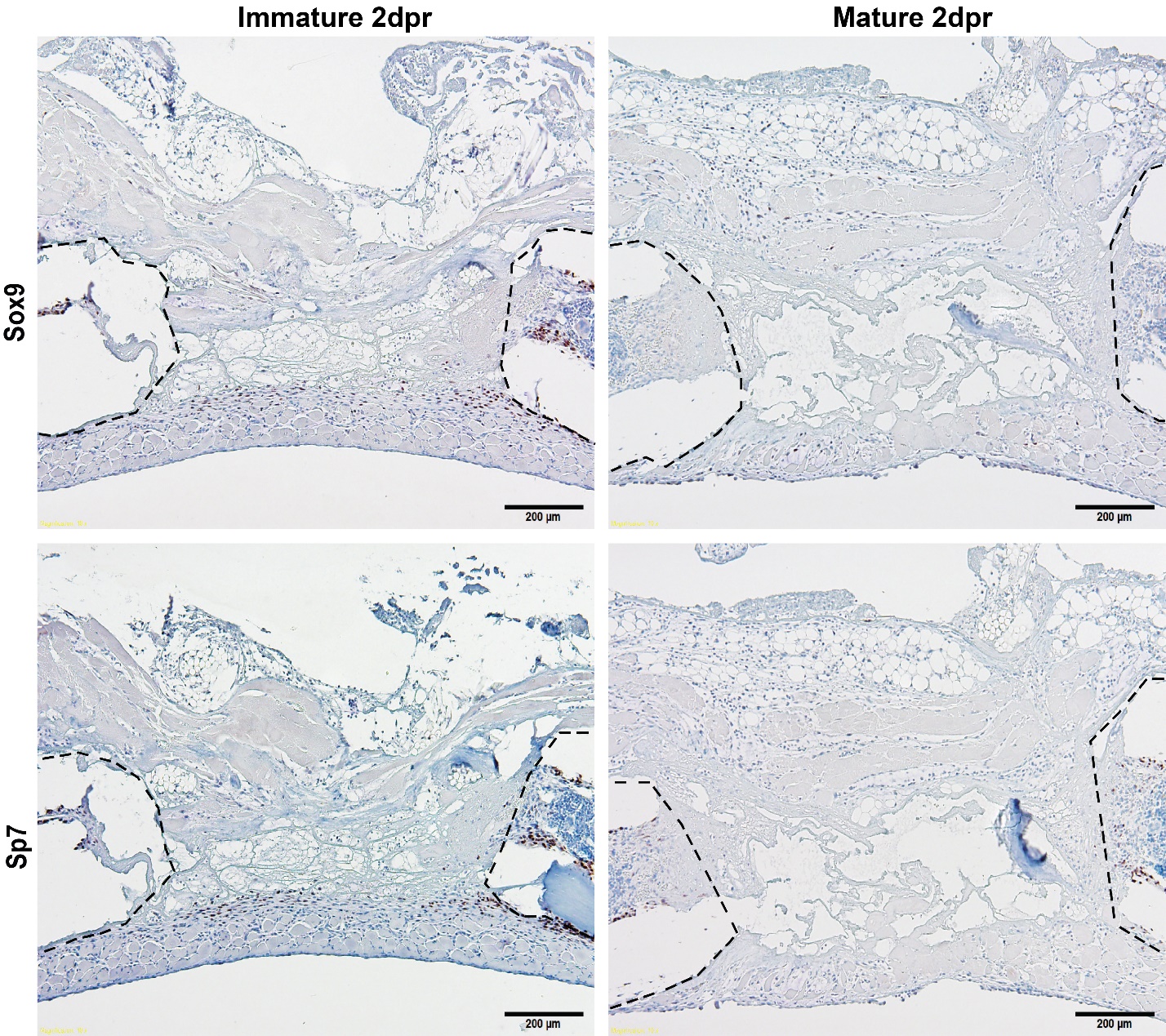
**

**Figure S2** The expression of SOX9 and SP7 are associated with skeletal maturity and age-related outcomes of large-scale rib defect repair. Immunohistochemical representation of SOX9^+^ and SP7^+^ cells in the repair calluses of immature and mature mice at 2dpr. The dashed black lines represent the ends of the ribs after resection surgery. Scale bars: 200μm.

**
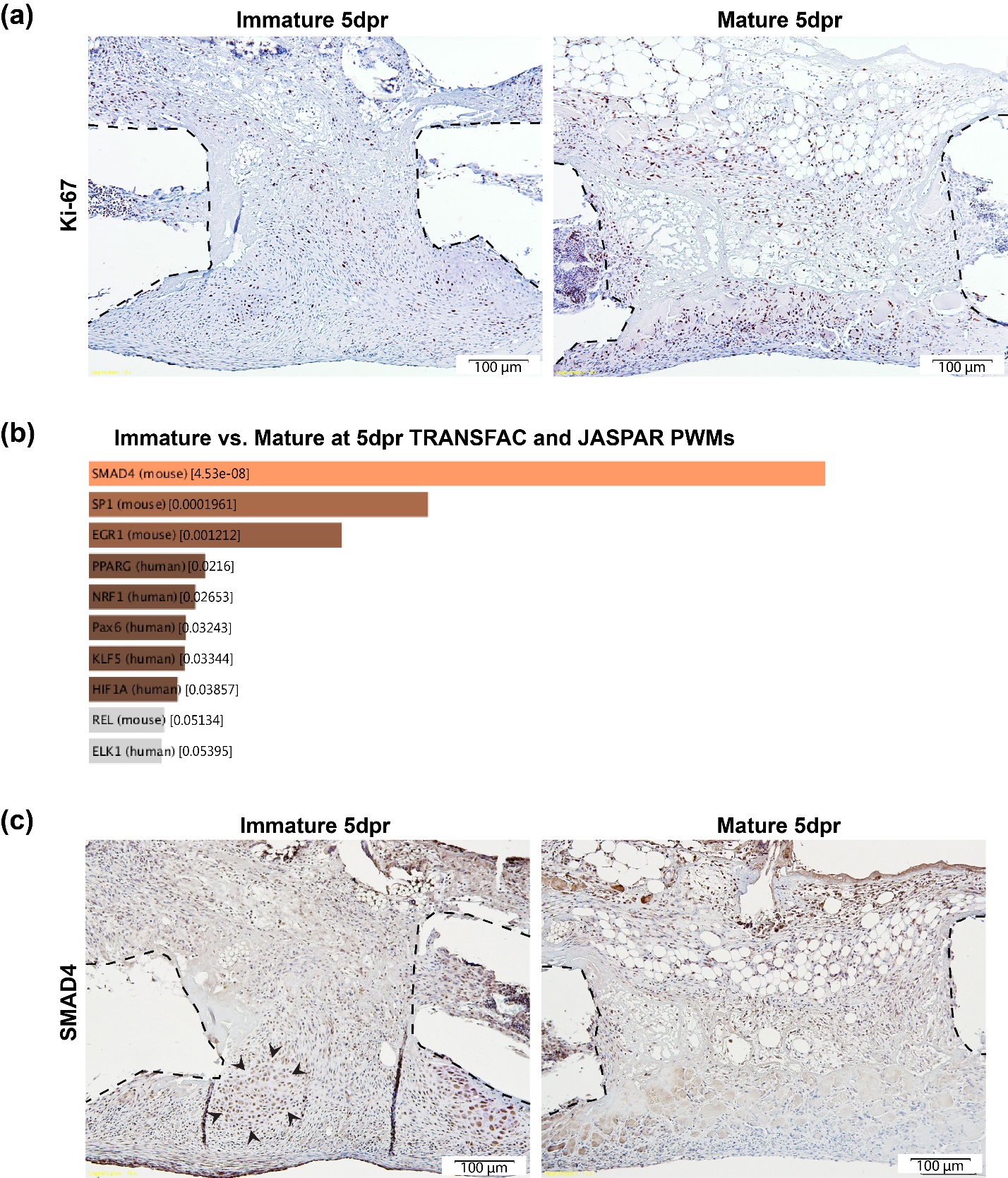
**

**Figure. S3** Assessment of Ki-67 and SMAD4 expression during the early-stage repair of large-scale rib defects. (**a**) Immunohistochemical staining of Ki-67 in the repair calluses of immature and mature mice at 5dpr. Scale bars: 100μm. (**b**) Bar graph represents the top 10 transcription factor-enriched terms of differentially expressed genes from Sox9-expressing cells of the osteochondrogenic cluster in immature versus mature mice at 5dpr ranked by P-value using TRANSFAC and JASPAR PWMs database analysis (Table S2 and Data S1). (**c**) Immunohistochemical representation of SMAD4^+^ cells in the repair calluses of immature and mature mice at 5dpr. Black arrowheads indicate the area of hypertrophic chondrocyte differentiation. The dashed black lines represent the ends of the ribs after resection surgery. Scale bars: 100μm.

**
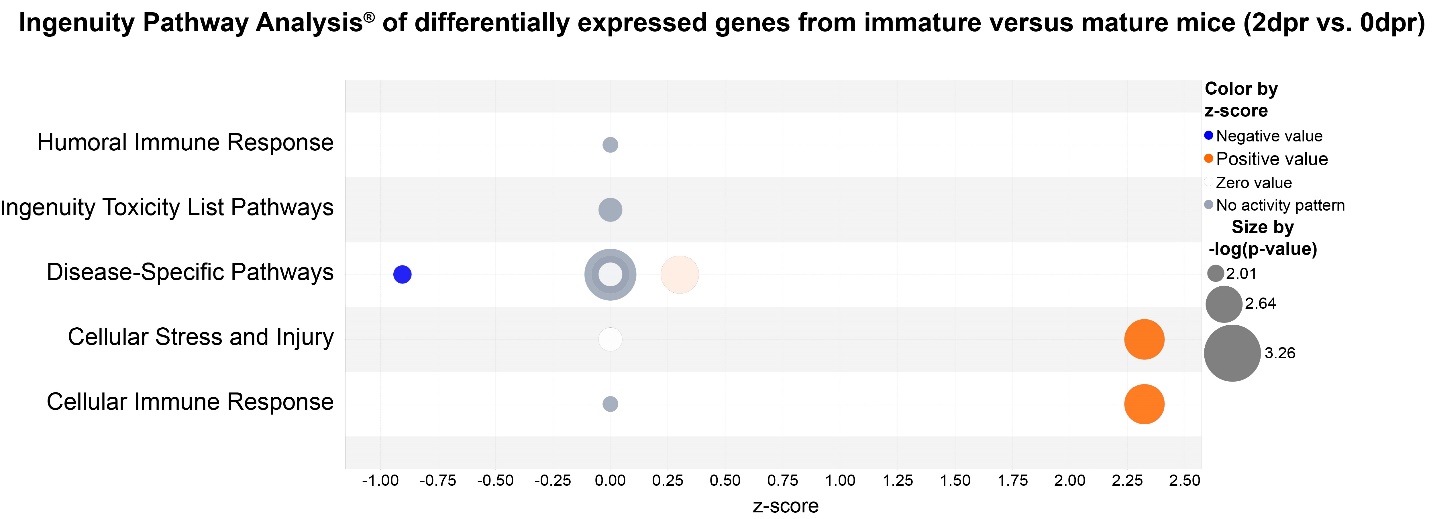
**

**Figure. S4** Bubble plot of differentially expressed genes between the calluses of immature and mature mice after rib resection. The graph shows a significant enrichment of canonical pathways related to cellular stress, injury, and immune response. The activation or inhibition state of a given pathway was determined using z-scores. The size of the bubbles is proportional to -log(P-values). Gene expression analysis was performed at 2 versus 0dpr and obtained by Ingenuity Pathway Analysis (IPA) of bulk RNA sequencing data (Data S2-S3).

**
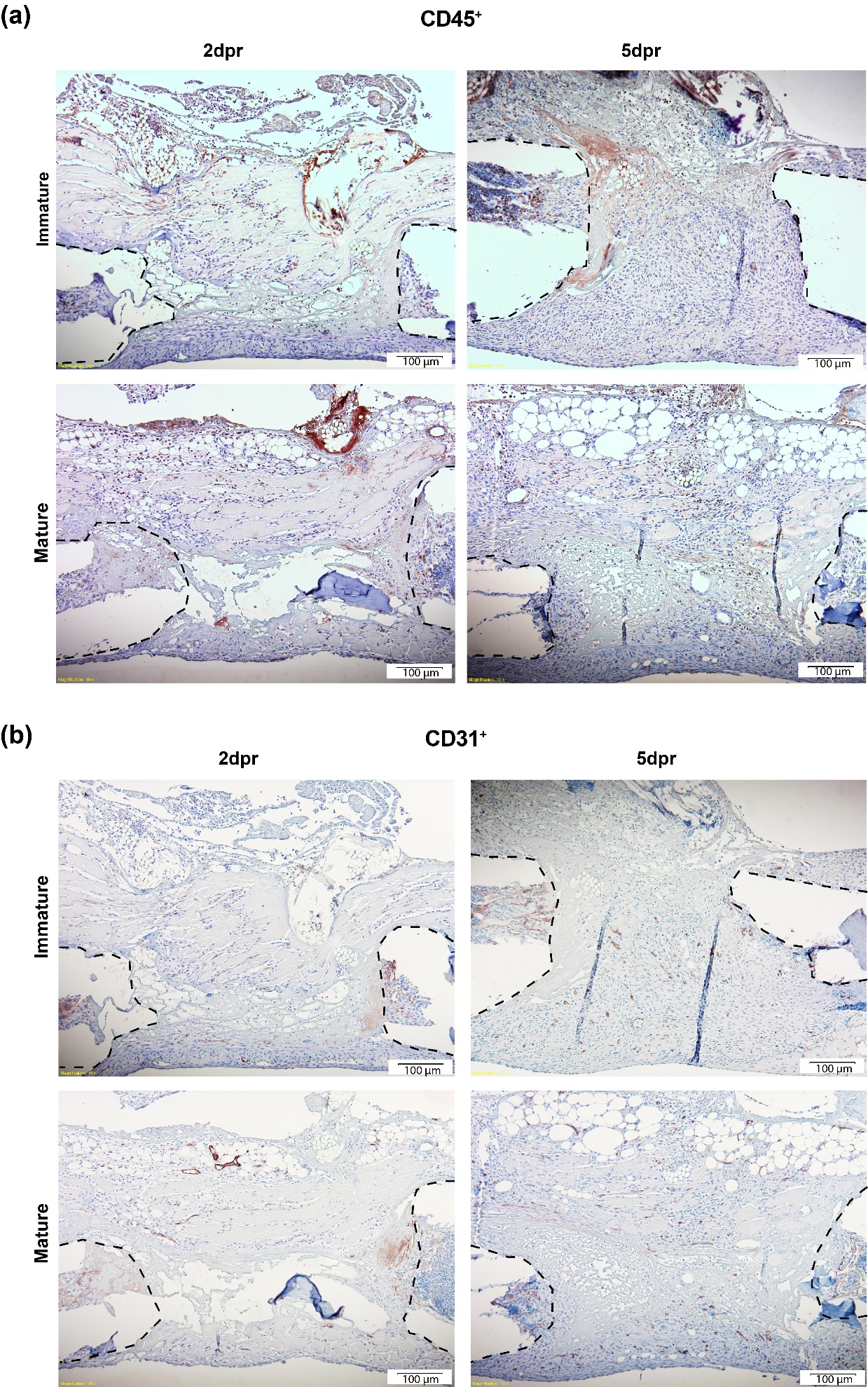
**

**Figure. S5** Differential spatial distribution of immune and endothelial cells in the callus of immature and mature mice. Representative immunohistochemical images of the distribution of CD45^+^ immune cells (**a**) and CD31^+^ endothelial cells lining the interior surface of blood vessels (**b**) in the repair calluses of immature and mature mice at 2dpr and 5dpr. The dashed black lines represent the ends of the ribs after resection surgery. Scale bars: 100μm.


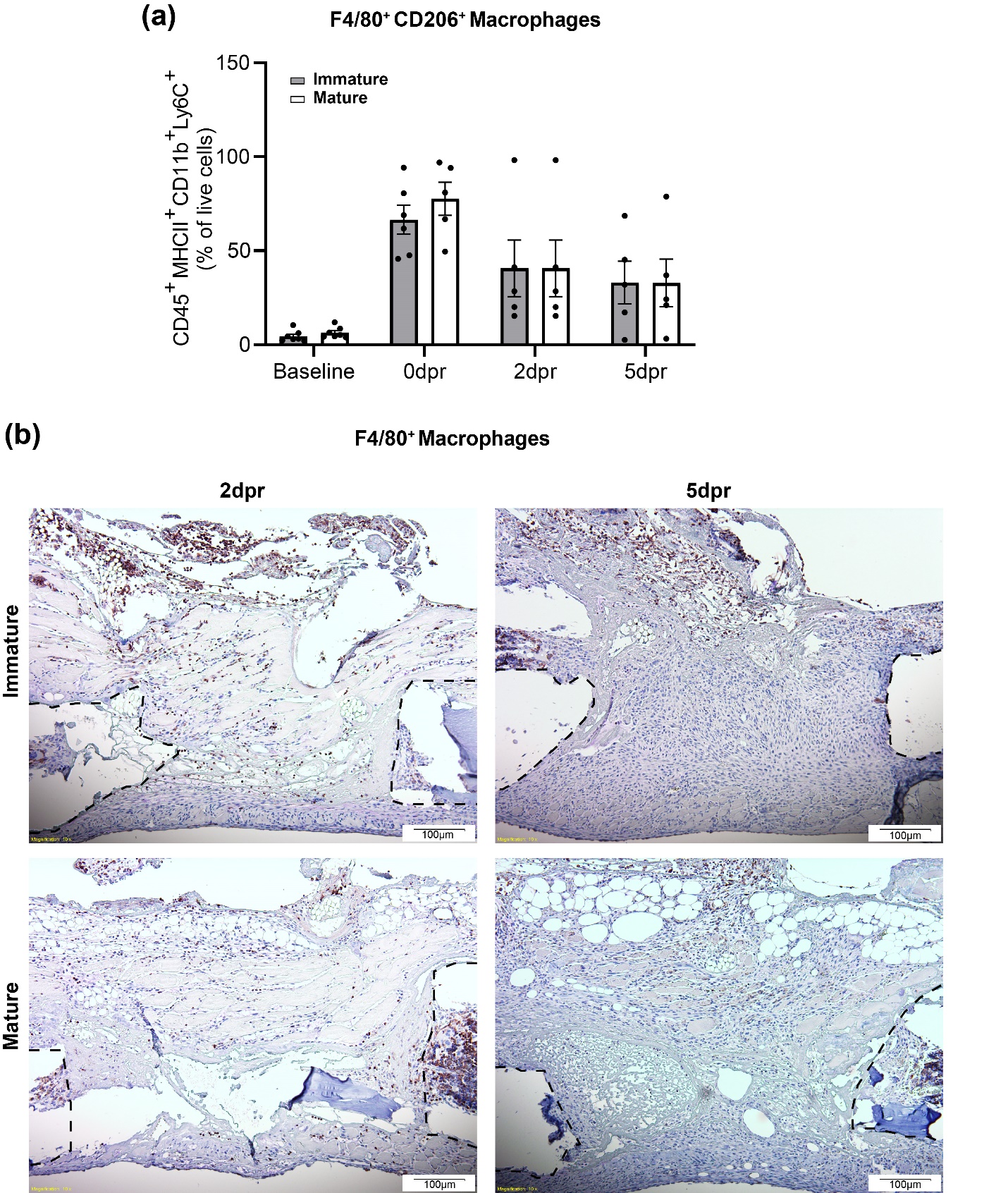


**Figure. S6** Skeletal maturity and age-related features of macrophage callus infiltration. (**a**) Flow cytometric analysis of the percentage of macrophages (CD11b^+^MHCII^+^Ly6C^+^CD11c^-^F4/80^+^) expressing CD206 in the rib calluses of immature and mature mice at baseline and during the early stages of repair (0, 2, and 5dpr). The gate strategy was defined as shown in Figure S13. Unpaired Student’s t test was used to find differences between immature and mature mice groups. Each dot represents a data point from an individual mouse, and bars depict means ±SEM (n= 5 or more mice/group). (**b**) Immunohistochemical representation of F4/80^+^ macrophages in the repair calluses of immature and mature mice at 2 and 5dpr. The dashed black lines represent the ends of the ribs after resection surgery. Scale bars: 100μm.

**
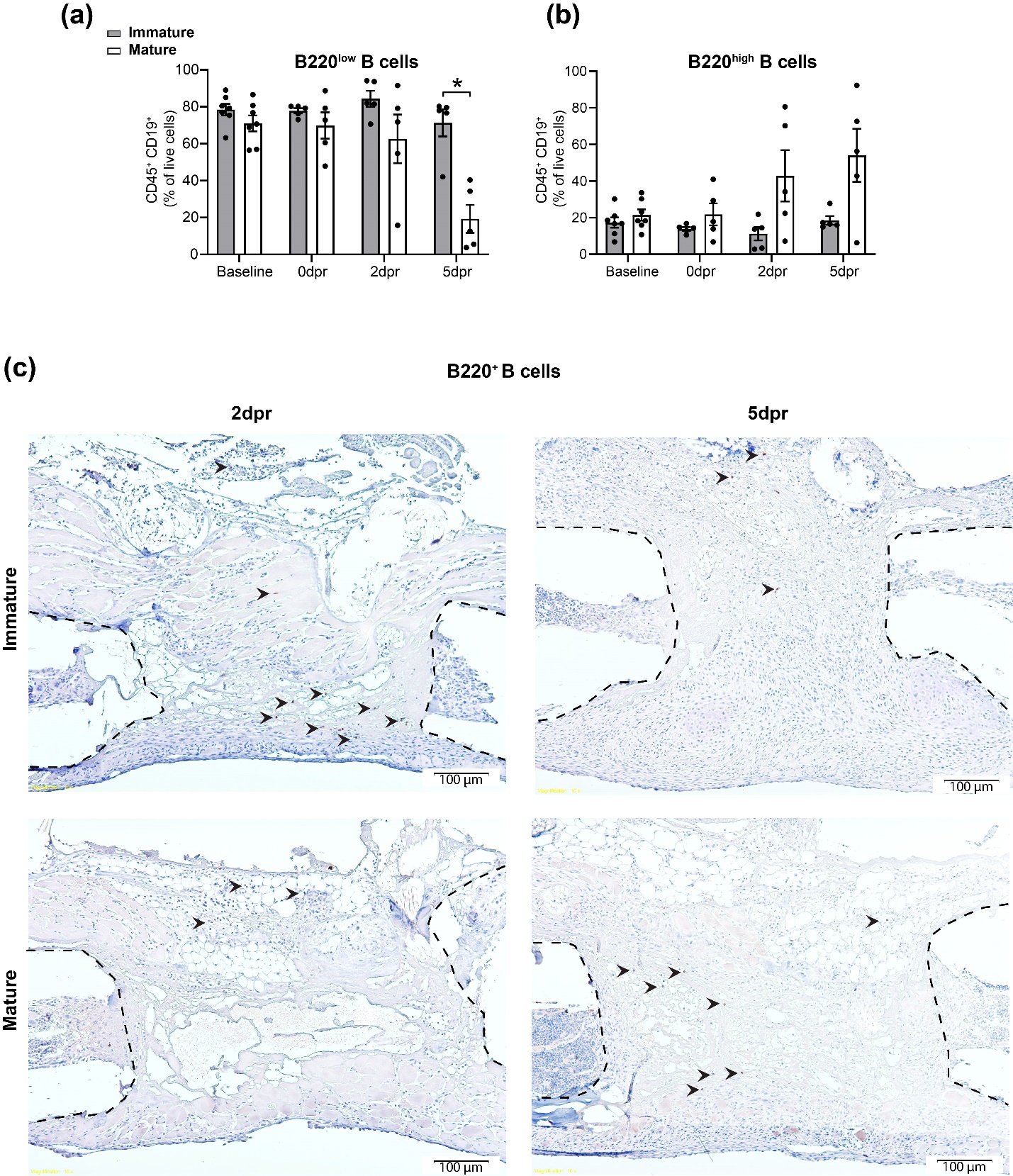
**

**Figure S7** Skeletal maturity and age alters the infiltration of B cells during the repair of large-scale rib defects. Flow cytometric analysis of the percentage of CD19^+^B220^low^ B cell subtype (**a**) was significantly reduced at 5dpr (*P < 0.05), while the CD19^+^B220^high^ B cell subtype (**b**) showed a tendency to increase at 2 (P = 0.05) and 5dpr (P = 0.15) in the rib calluses of mature mice. The gate strategy was defined as shown in Figure S13. Unpaired Student’s t test was used to find differences between immature and mature mice groups. Each dot represents a data point from an individual mouse, and bars depict means ±SEM (n = 5 or more mice/group). (**c**) Representative immunohistochemical images of B220^+^ B cells in the repair calluses of immature and mature mice at 2 and 5dpr. The dashed black lines represent the ends of the ribs after resection surgery. Scale bars: 100μm.

**
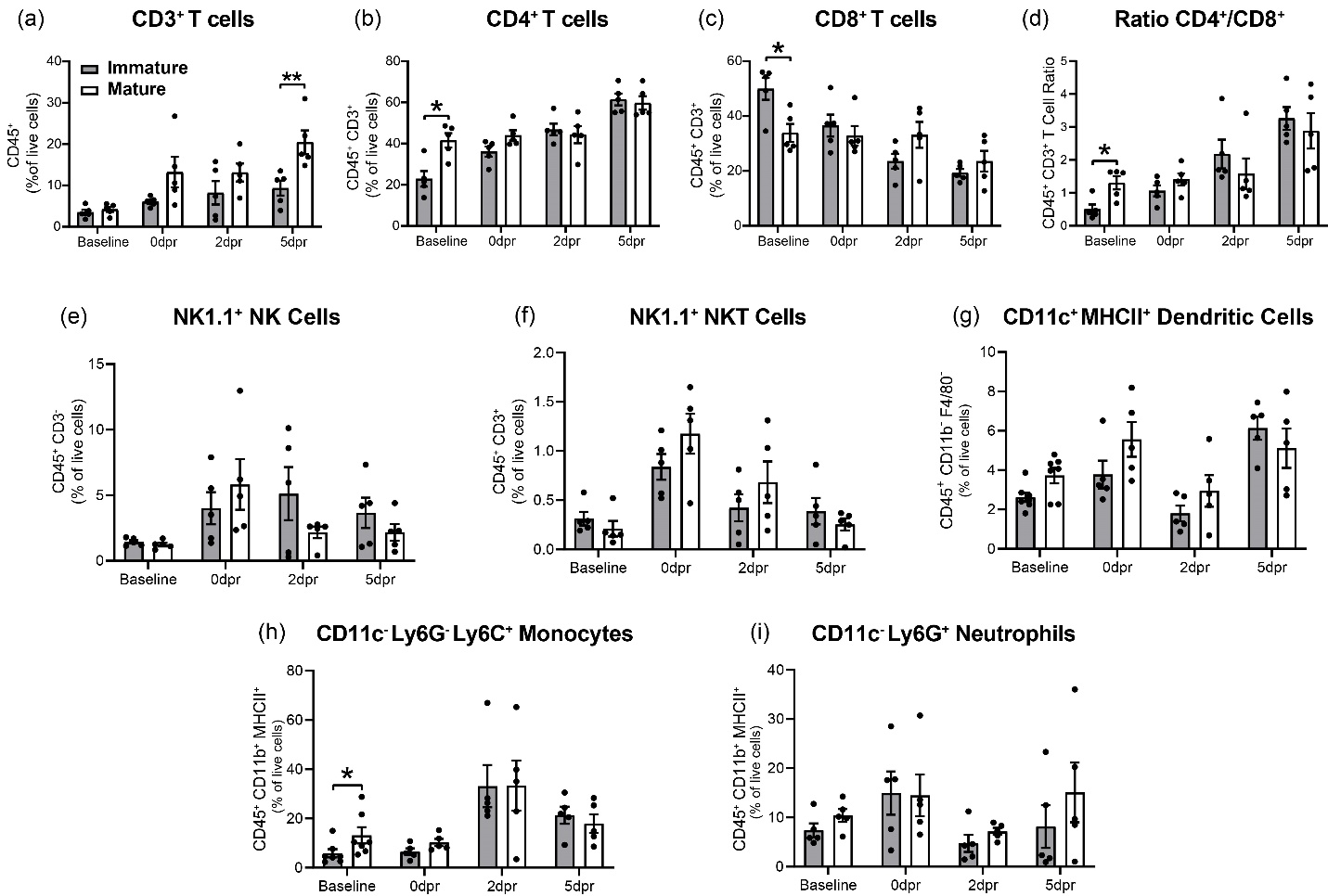
**

**Figure S8** Flow cytometric analysis of immune cells in rib calluses. Bar graph represents the percentage of total CD3^+^ T cells (**a**), CD3^+^CD4^+^ T cells (**b**), CD3^+^CD8^+^ T cells (**c**), T CD4^+^/CD8^+^ ratio (**d**), CD3^-^NK1.1^+^ NK cells (**e**), CD3^+^NK1.1^+^ NKT cells (**f**), CD11c^+^MHCII^+^CD11b^-^F4/80^-^ dendritic cells (**g**), CD11b^+^MHCII^+^CD11c^-^Ly6G^-^Ly6C^+^ monocytes (**h**), and CD11b^+^MHCII^+^CD11c^-^Ly6G^+^ neutrophils (**i**) in the rib calluses of immature and mature mice at baseline and during the early stages of repair (baseline, 0, 2, and 5dpr). The gate strategy was defined as shown in Figure S13 Figure. Unpaired t test was used to find differences between immature and mature mice groups. Each dot represents a data point from an individual mouse, and bars depict means ±SEM (*p< 0.05, **p< 0.01; n = 5 or more mice/group).

**
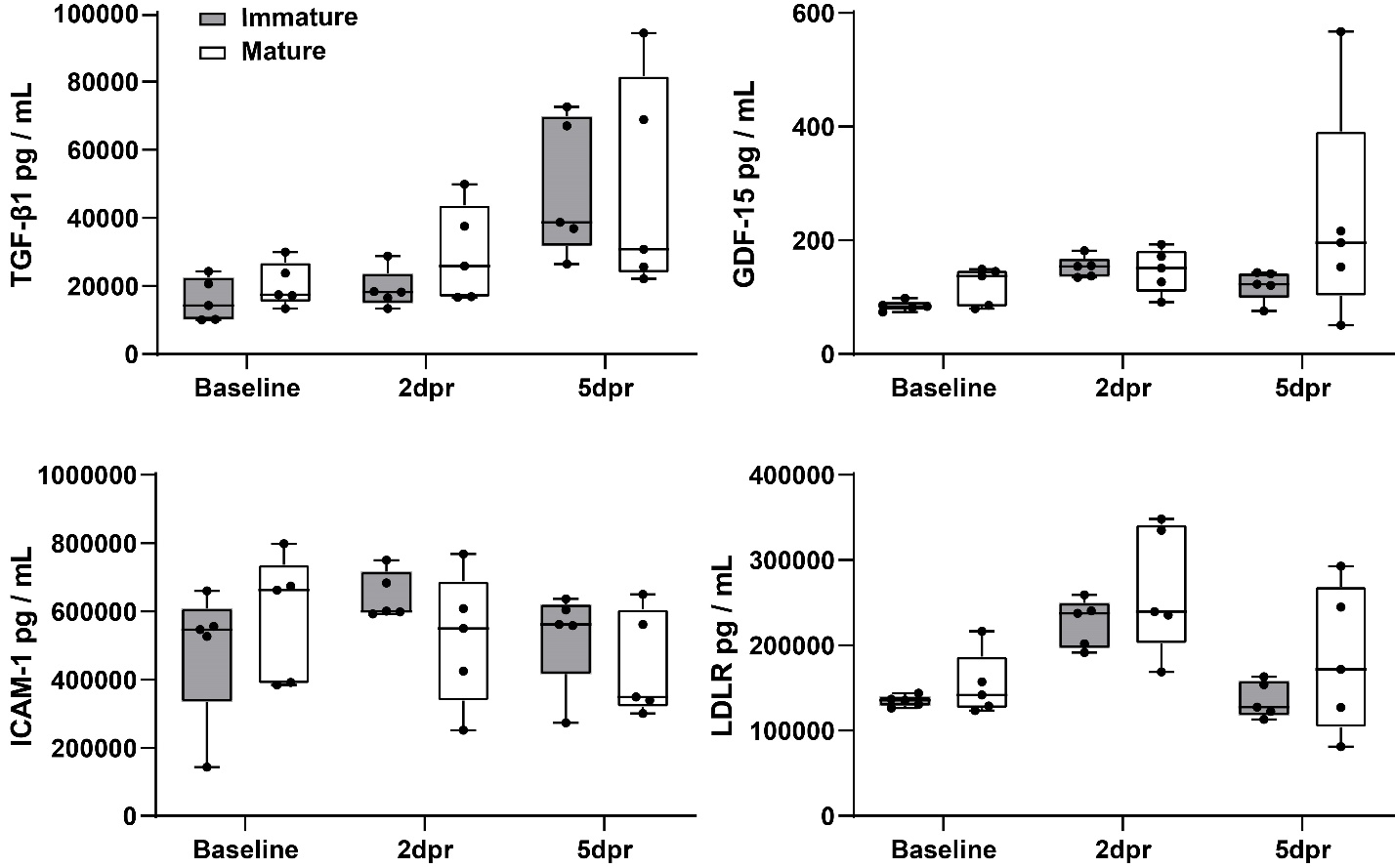
**

**Figure S9** Plasma levels of circulatory factors before and after rib resection. No significant differences were found in the concentrations (pg/mL) of TGF-β1, intercellular adhesion molecule 1 (ICAM-1), growth differentiation factor-15 (GDF-15), and soluble low-density lipoprotein receptor (LDLR) measured by Luminex between immature and mature mice at baseline, 2, and 5dpr (n = 5 mice/group). Each dot represents a data point from an individual mouse, and boxes represent the minimum, median, and maximum values. All samples had P < 0.05 and comparisons were analyzed using the unpaired Student’s t test for each time point. Basic fibroblast growth factor (FGF-basic), IL-1α, IL-1ra, IL-3, IL-5, IL-18, IL-27, Epidermal growth factor (EGF), and IL-17 were only detected in isolated samples. IL-2, IL-4, IL-7, IL-9, IL-10, IL-11, IL-12p70, IL-13, IL-21, IL-31, granulocyte-macrophage colony-stimulating factor (GM-CSF), and interferon- γ (IFN-γ) were not detected at any time point in any of the samples.

**
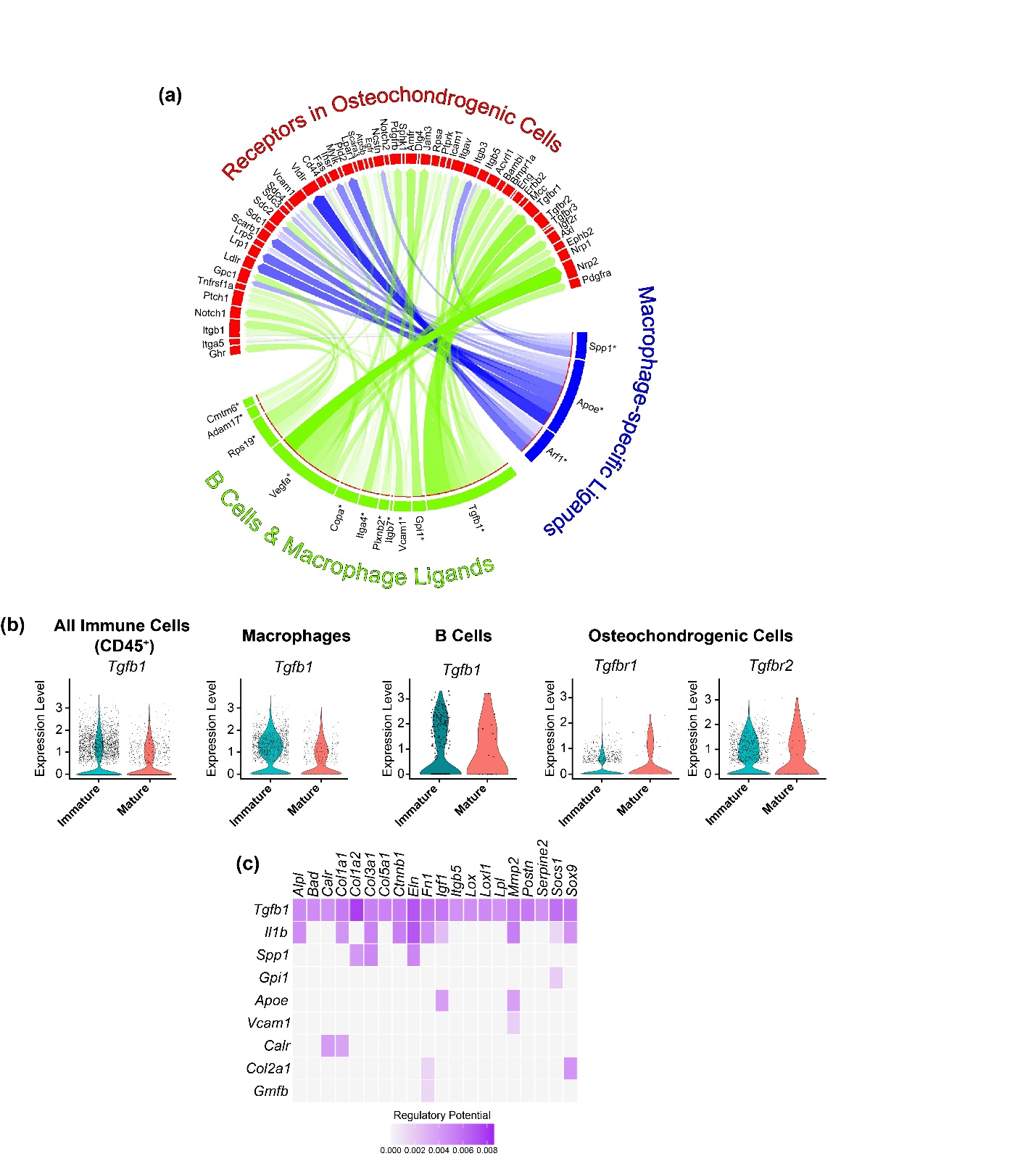
**

**Figure S10** Crosstalk between immune and osteochondrogenic cells may play an important role during rib regeneration. (**a**) Circos plot displaying ligand-receptor interactions between macrophages/B cells and osteochondrogenic cells captured at 5dpr. Overall, network analysis mainly predicted cellular interactions involving components of TGF-β signaling (Tgfb1-Tgfbr1/2) and other pathways related to bone regeneration (such as Spp1-Itgb3/5, and Spp1-Ncstn), skeletal tissue mineralization (Gpi1-Amfr), cartilage and bone formation (Il1b-Acan/Bgn/Col1a1/Col2a1/Scx/Tnmd), lipid metabolism (such as Apoe-Ldlr, Apoe-Vldlr, Apoe-Lrp1/5, and Arf1-Pdl2), and angiogenesis (such as Vegfa-Nrp1/2, and Vegfa-Pdgfra). (**b**) Violin plots represent the relative expression of the gene *Tgfb1* that codifies the TGF-β1 ligand in *Ptprc*^+^ immune cells, *Adgre1*^+^ macrophages, or *Cd19*^+^ B cells, and its corresponding receptors *Tgfbr1/2* in osteochondrogenic cells at 5dpr. (**c**) Heatmap of scaled expression of ligands in macrophages and B cells, averaged per target gene in osteochondrogenic cells identified by NicheNet analysis. Clusters were generated using a resolution of 0.1. Expression values are normalized and scaled averages.

**
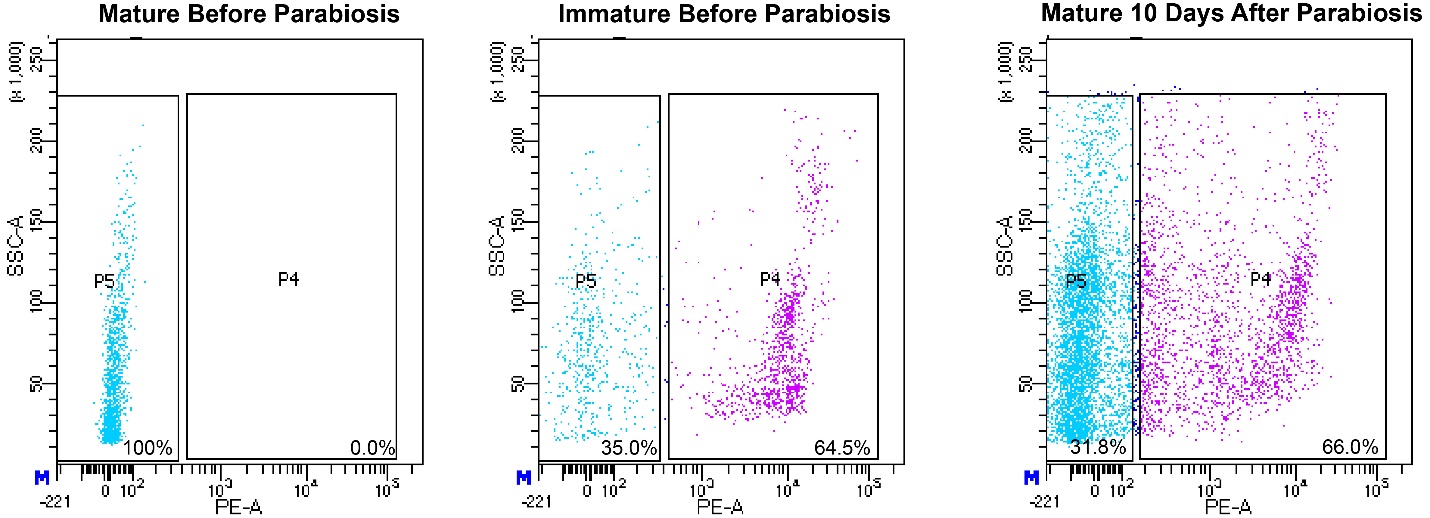
**

**Figure S11** Flow cytometric analysis of peripheral blood from mice before and after parabiosis. Representative images obtained by flow cytometric analysis show tdTomato^-^ (blue – P5) and tdTomato^+^ (pink – P4) cells in the peripheral blood of mature and immature mice, respectively, before parabiosis. We confirmed blood-sharing between parabiont pairs by observing tdTomato^+^ cells in the circulation of both mice 10 days after parabiosis.

**
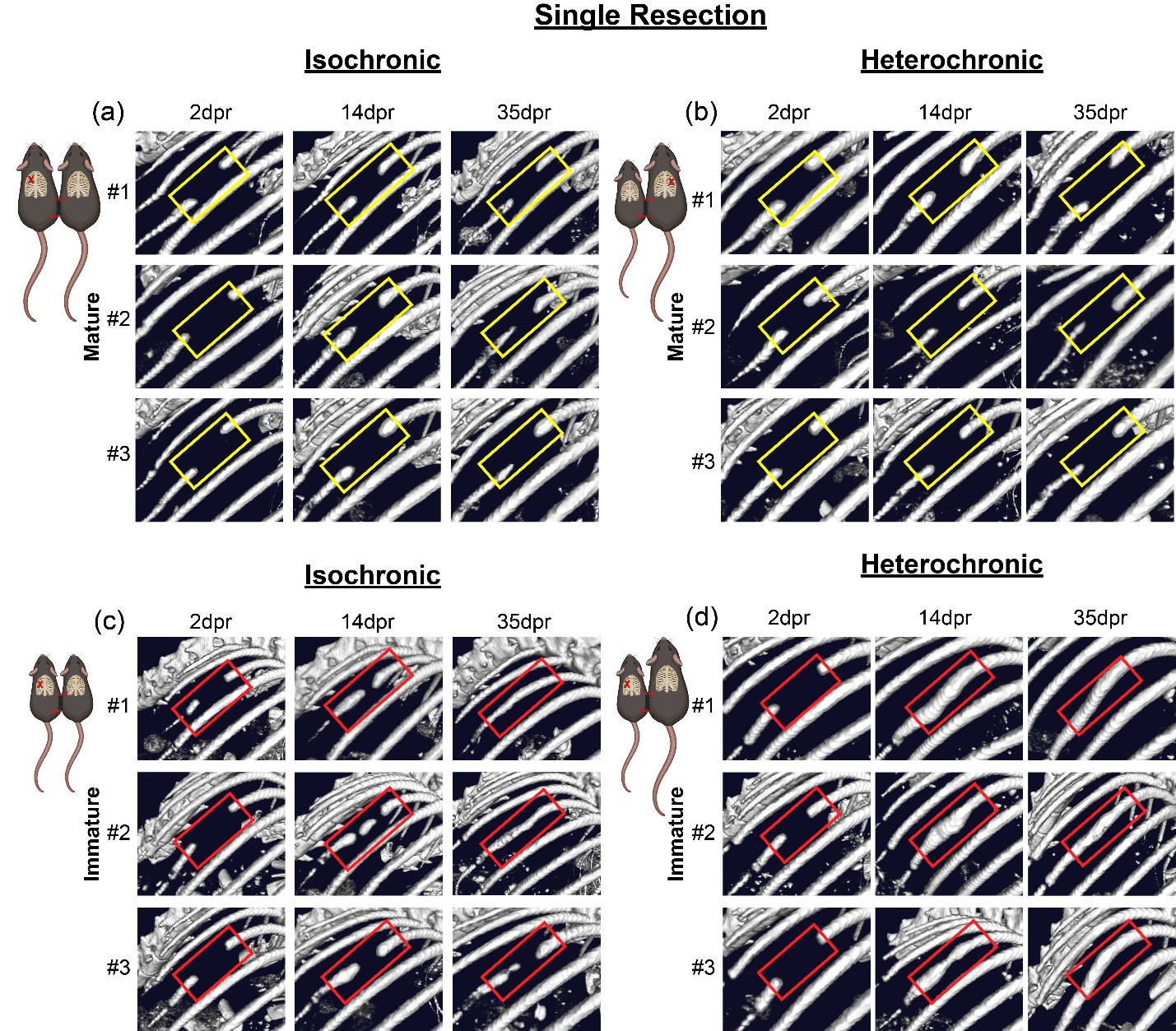
**

**Figure S12** Live µCT analysis of rib repair outcomes in single-resected pairs of mice under isochronic and heterochonic parabiosis. µCT live images of large-scale bone repair outcomes at 2, 14, and 35dpr in: (**a**) mature mice under isochronic parabiosis; (**b**) mature mice under heterochronic parabiosis; (**c**) immature mice under isochronic parabiosis; (**d**) immature mice under heterochronic parabiosis. Only one animal in each pair underwent rib resection (n = 3/group). The red and yellow rectangles highlight areas of the ribs that were surgically resected and followed for any sign of bone regeneration from 2 to 35dpr in immature and mature mice, respectively. Cartoons were created with BioRender.com, showing illustrative images of relatively small and large mice representing immature and mature mice, respectively, under isochronic or heterochronic parabiosis. The red “X” on the chest of one mouse in each illustrated parabiont pair represents rib resection.

**
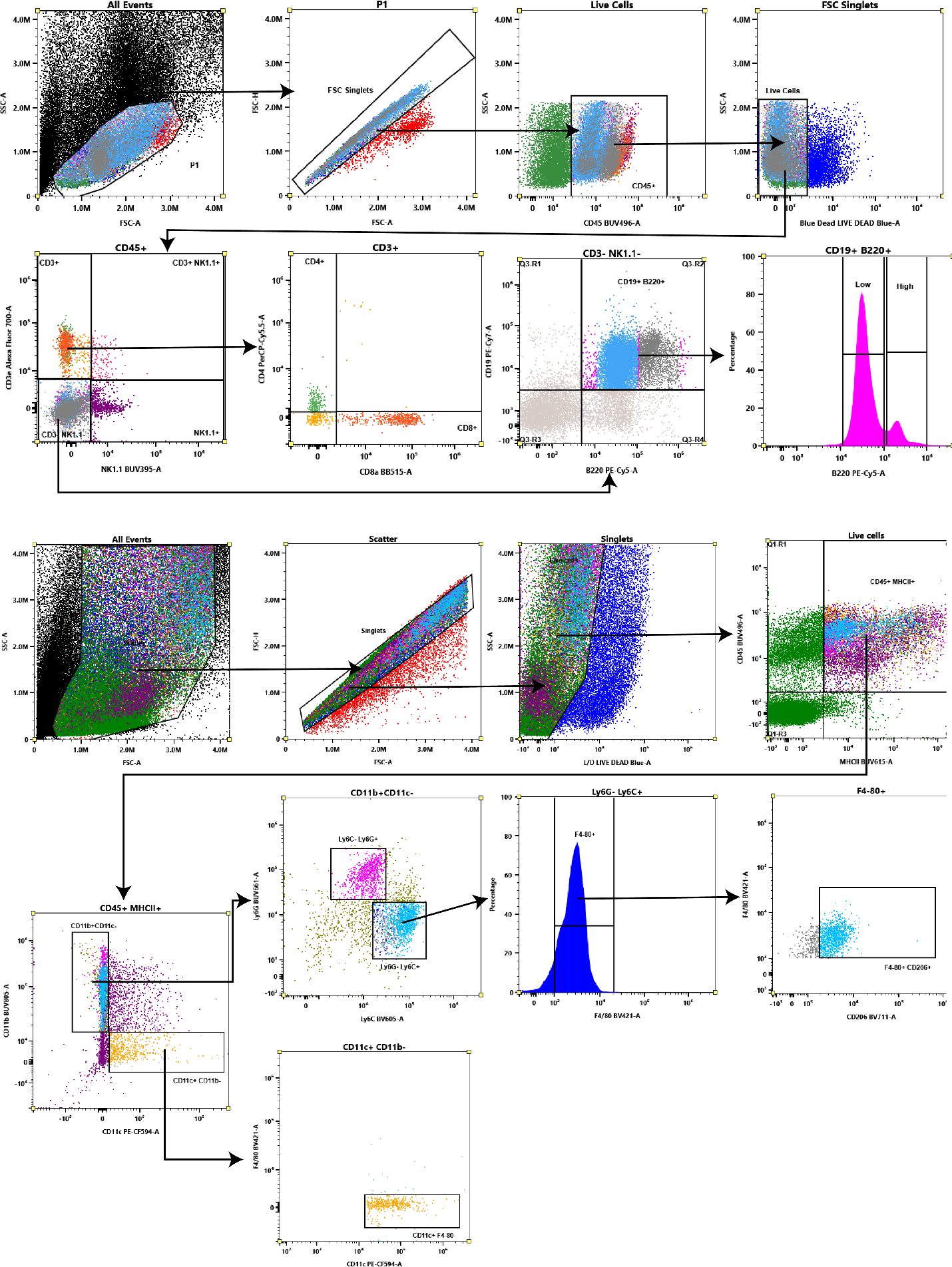
**

**Figure S13** Gating strategies for flow cytometry data analysis. Representative dot-plot and histogram charts were used to define different immune cell subpopulations (CD45^+^) such as NK cells (CD3^-^NK1.1^+^), NKT cells (CD3^+^NK1.1^+^), T cells (CD3^+^), B cells (CD3^-^ NK1.1^-^CD19^+^B220^+^), macrophages (CD11b^+^MHCII^+^Ly6C^+^CD11c^-^F4/80^+^), monocytes (CD11b^+^MHCII^+^CD11c^-^Ly6G^-^Ly6C^+^), neutrophils (CD11b^+^MHCII^+^CD11c^-^Ly6G^+^), and dendritic cells (CD11c^+^MHCII^+^CD11b^-^F4/80^-^). Dead cells were excluded with Blue Fixable Live/Dead cell dye. Lymphocytes and myeloid cells were gated based on SSC-A versus FSC-A, and singlets were selected from the FSC-A versus FSC-H dot plots. Samples were gated for specific cell populations based on Fluorescence Minus One (FMO) controls.

**Table S1** Age, sex and volume of the patient’s regenerated ribs

| Patient # | Age | SEX | % REGENERATED BONE Volume  (6 months AFTER SURGERY) |
| --- | --- | --- | --- |
| 1 | 5 | Female | 109.5 |
| 2 | 12 | Male | 127.5 |
| 3 | 14 | Male | 113.6 |
| 4 | 14 | Male | 69 |
| 5 | 16 | Male | 126.7 |
| 6 | 16 | Female | 100.4 |
| 7 | 20 | Male | 70.7 |
| 8 | 25 | Female | 66.4 |
| 9 | 38 | Female | 92.5 |
| 10 | 42 | Male | 34 |
| 11 | 45 | Male | 18.9 |

**Table S2** Cluster defining genes.

**Table S3** Enrichment analysis of upregulated genes in *Sox9*-expressing cells (from cluster #2).

**Table S4** List of soluble target analytes evaluated in plasma samples from immature and mature mice using the Mouse XL Cytokine Luminex® Performance Premixed Kit (#FCSTM20, R&D Systems, USA) and the TGF-beta 1 Magnetic Luminex® Performance Assay (#LTGM00, R&D Systems, USA)

| TARGET ANALYTE | RANGE OF STANDARD CONCENTRATION (pg/mL) |
| --- | --- |
| BAFF/BLyS | 22.4 – 16,561.15 |
| FGF-basic | 11.72 – 8,891.75 |
| GDF-15 | 17.75 – 12,969.87 |
| ICAM-1 | 12,764.89 – 9.45 x 10^6^ |
| IL-1a | 35,09.03 – 23,199.93 |
| IL-1Ra | 75.13 – 55,935.18 |
| IL-3 | 74.86 – 6,515.62 |
| IL-5 | 14.84 – 11,250.39 |
| IL-7 | 147.12 – 110,871.5 |
| IL-10 | 35.82 – 26,227.11 |
| IL-12p70 | 83.76 – 61,208.05 |
| IL-16 | 84.33 – 63,236.35 |
| IL-18 | 41.08 – 33,522.62 |
| IL-27 | 11.38 – 10,024.81 |
| LDL-R | 1,247.53 – 932,827.77 |
| EGF | 10.67 – 8,144.21 |
| G-CSF | 209.28 – 151,193.24 |
| GM-CSF | 19.76 – 14,650.3 |
| IFN-g | 11.94 – 8,928.87 |
| IL-1b | 23.84 – 17,612 |
| IL-2 | 5.06 – 3,890.74 |
| IL-4 | 67.51 – 50,673.03 |
| IL-6 | 16.34 – 12,525.72 |
| IL-9 | 7.63 – 5,722.9 |
| IL-11 | 29.52 – 22,906.06 |
| IL-13 | 104.67 – 86,324.15 |
| IL-17 | 24.15 – 18,282.91 |
| IL-21 | 22.23 – 15,922.64 |
| IL-31 | 77.38 – 56,071.22 |
| M-CSF | 4.95 – 3,615.72 |
| TGF-b1 | 25.23 – 25,089.49 |

**Table** **S5** List of antibodies used for immunohistochemistry.

**
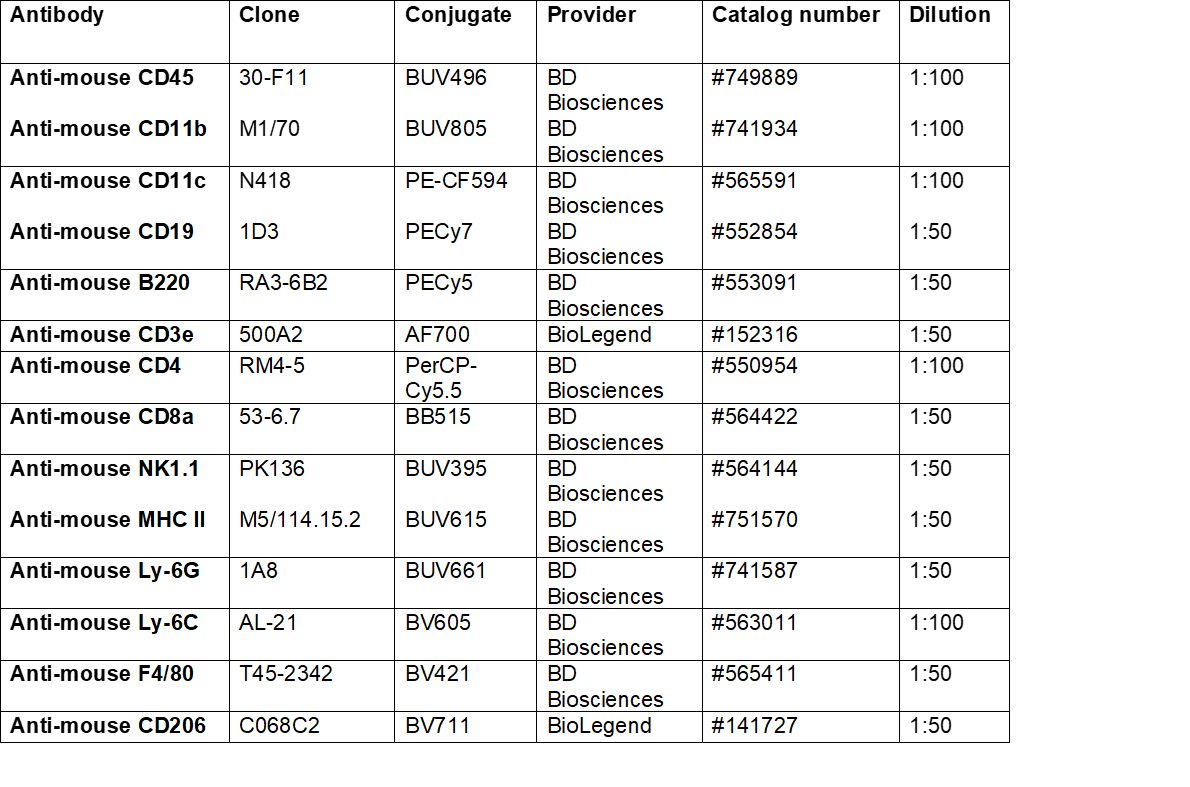
Table S6** List of antibodies used for flow cytometry assays.

**Reference:**

Lu, J., Doyle, A. D., Shinsato, Y., Wang, S., Bodendorfer, M. A., Zheng, M., & Yamada, K. M. (2020). Basement Membrane Regulates Fibronectin Organization Using Sliding Focal Adhesions Driven by a Contractile Winch. *Dev Cell*, *52*(5), 631-646.e634. <https://doi.org/10.1016/j.devcel.2020.01.007>
